# Mesenchymal stem cell-derived extracellular vesicles protect retina in a mouse model of retinitis pigmentosa by anti-inflammation through miR-146a-Nr4a3 axis

**DOI:** 10.1186/s13287-022-03100-x

**Published:** 2022-08-03

**Authors:** Jia Zhang, Pengdong Li, Guifang Zhao, Siqi He, Di Xu, Weijie Jiang, Qian Peng, Zhaohui Li, Zhongjian Xie, Han Zhang, Ying Xu, Ling Qi

**Affiliations:** 1 The Sixth Affiliated Hospital of Guangzhou Medical University, Qingyuan People’s Hospital, B24 Yinquan South Road, Qingyuan, 511518 Guangdong China; 2 Guangdong-Hong Kong-Macau Institute of Central Nervous System Regeneration, Key Laboratory of Central Nervous System Regeneration, Ministry of Education, Jinan University, Guangzhou, 510632 China; 3 College of Basic Medicine, Beihua University, Jilin City, 132013 Jilin China; 4 Institute of Pediatrics, Shenzhen Children’s Hospital, Shenzhen, 518038 China; 5 Shenzhen Engineering Laboratory of Phosphorene and Optoelectronics, International Collaborative Laboratory of 2D Materials for Optoelectronics Science and Technology of Ministry of Education, Shenzhen University, Shenzhen, 518060 China; 6 The Key Laboratory of Pathobiology, Department of Pathology, College of Basic Medical Sciences, Jilin University, Ministry of Education, Changchun, 130021 China; 7 School of Basic Medicine, Gannan Medical University, Ganzhou, 341000 China

**Keywords:** Retinitis pigmentosa, rd10 mouse, Extracellular vesicles, Inflammation, miR-146a-Nr4a3 axis

## Abstract

**Background:**

Retinitis pigmentosa is a rod-cone degenerative disease that induces irreversible vision loss. This study probed the protective capacity of mesenchymal stem cell-derived small EVs (MSC-EVs) on the retinas of rd10 mice and the underlying mechanism.

**Methods:**

MSC-EVs were injected into the vitreous of rd10 mice at postnatal day 14 and P21; morphology and function were examined at P28. The mechanism of action was explored by using co-culture of photoreceptor cell line 661 W and microglia cell line BV2.

**Results:**

Treatment with MSC-EVs increased the survival of photoreceptors and preserved their structure. Visual function, as reflected by optomotor and electroretinogram responses, was significantly enhanced in MSC-EVs-treated rd10 mice. Mechanistically, staining for Iba1, GFAP, F4/80, CD68 and CD206 showed that MSC-EVs suppressed the activation of microglial, Müller glial and macrophages. Furthermore, western blotting showed that the treatment inhibited the NF-κB pathway. RNA-seq and qPCR showed that MSC-EVs upregulated anti-inflammatory cytokines while downregulating pro-inflammatory cytokines. MSC-EVs application in vitro decreased the number of TUNEL-positive 661 W cells co-cultured with LPS-stimulated BV2, with similar impact on the cytokine expression as in vivo study. Genetic screening predicted miR-146a to be the downstream target of MSC-EVs, which was detected in MSC-EVs and upregulated in co-cultured 661 W cells and BV2 cells after MSC-EVs treatment. Upregulation of miR-146a by using its mimic decreased the expression of the transcription factor Nr4a3, and its downregulation inhibition promoted Nr4a3 expression in both 661 W and BV2 cells. Nr4a3 was further identified as the target gene of miR-146a by dual-luciferase assay. Furthermore, overexpressing miR-146a significantly decreased the expression of LPS-induced pro-inflammatory cytokines in BV2 cells.

**Conclusions:**

MSC-EVs delays retinal degeneration in rd10 mice mainly by its anti-inflammatory effect via the miR-146a-Nr4a3axis. Hence, MSC-EVs may be used in the treatment of neurodegenerative diseases.

**Supplementary Information:**

The online version contains supplementary material available at 10.1186/s13287-022-03100-x.

## Introduction

The retina is an important part of the central nervous system, and its retinopathies always lead to irreversible vision loss. For example, retinitis pigmentosa (RP) is a familial disease that affects approximately 1 in 4000 people worldwide [1]. This disease is characterized by the death of rod photoreceptors followed by cone photoreceptors [2]. Although we have a profound understanding of the etiopathogenesis of RP, there is still no effective treatment. One of the main pathologies during photoreceptor degeneration is the inflammatory microenvironment around the photoreceptors [3]. Treatment with anti-inflammatory components such as LBP (*Lycium barbarum* polysaccharides) [4, 5] has been proved to be effective in protecting retinas and slowing down photoreceptor degeneration or by agents blocking microglial adenosine [6], silencing *IL2rg* [7], etc. Therefore, inhibiting inflammation has a strong potential in clinical application to treat retinal degenerative diseases.

Stem cell-based therapies have been applied in the clinic trial to treat retinal diseases with promising results [8], and they act by supporting the diseased cells via cellular autonomy, or by modulating immunity and inflammation to improve the retinal environment [8, 9]. However, many challenges remain, such as the production of clinical standard cells, transplantation, immune rejection, potential carcinogenic risk and unclear mechanisms [8, 10]. A potentially better approach is using extracellular vesicles (EVs) that are secreted from stem cells. Their advantage over stem cells has to do with their low immunogenicity and easier administration. EVs or exosomes (Exos, EVs with 30–200 nm diameter size) collected from mesenchymal stem cells (MSCs) have been applied to animal models of retinal and eye diseases including diabetic retinopathy (DR), glaucoma and age-related macular degeneration (AMD) and proved effective mainly through their powerful anti-inflammatory effects [11–15]. One study of a rat model used neural progenitor cells (NPC)-Exos and reported an inhibition of the pro-inflammatory factors (including tumor necrosis factor-α/TNF-α, IL-1β and COX-2) [9], but only few studies explored the effect and mechanism of MSC-EVs on degenerated photoreceptors. Whether the MSC-EVs can protect photoreceptor in a similar way, by inhibiting pro-inflammatory processes, and how it modulates the inflammatory responses remained unclear.

Here, we investigated whether MSC-EVs can delay photoreceptor degeneration in *Pde6b* mutant rd10 mice by suppressing inflammation and explored the mechanism. The rd10 mouse is a good model for human RP because it carries a homozygous missense mutation in the beta subunit of the rod phosphodiesterase gene *Pde6b* [16], a mutation that causes RP in humans [17]. In the rd10 mouse model, the death of rod photoreceptors begins around postnatal day (P) 17 and is nearly complete by P45 [18]. We show that a weekly intravitreal injection of MSC-EVs from P14 to P28 protects degenerated rods and cones, resulting in the improvement of visual performance. This neuroprotective effect acted mainly via miR-146a-Nr4a3 axis to modulate inflammation.

## Materials and methods

### Animals

Rd10 (*Pde6b*^*rd10*^) mice were purchased from the Jackson Laboratory (Bar Harbor, USA), and C57BL/6 J (wild-type, WT) control mice were purchased from Guangdong Medical Lab Animal Center (Guangdong, China). Littermate of rd10 mice was either treated with MSC-EVs or NS (normal saline) as the negative control group. All mice were raised under standard laboratory conditions with 12 h/12 h light/dark cycles and were fed with regular food and water. All animal experiments were conducted in accordance with the guidelines of National Institutes of Health Guide for the Care and Use of Laboratory Animals and approved by the ethics committees at the Sixth Affiliated Hospital of Guangzhou Medical University, Qingyuan People’s Hospital.

### Isolation of MSCs

Human umbilical cord MSCs were isolated from umbilical cord tissue, which was provided by the Clinical Biological Sample Bank of Qingyuan People’s Hospital. All experiments involving human tissues and cells were approved by the ethics committee of the ethics committees at the Sixth Affiliated Hospital of Guangzhou Medical University, Qingyuan People’s Hospital. Briefly, umbilical cord tissues were collected; segmented; suspended in Dulbecco’s modified Eagle medium containing nutrient mixture F-12 (DMEM/F-12), 10% fetal bovine serum (FBS), 1% penicillin/streptomycin and 10 ng/mL basic fibroblast growth factor (bFGF); and then maintained in a 37 °C humidified 5% CO_2_ incubator. The medium was replaced every three days before collecting cells at 1–2 weeks later, when most remaining cells were MSCs.

### Identification of MSCs by flow cytometry and immunofluorescence

To identify and isolate MSCs, cells were decomposed as single cells after hatching with FITC-conjugated primary antibodies against CD34, CD44, CD45, CD73, CD90 and CD105 (1:100, Thermo Fisher Scientific). Then, cells were incubated with the anti-rabbit or anti-mouse secondary antibody (Alexa-488, 1:1000, CST). Immunofluorescence was applied to observe the expression of CD34, CD44, CD45, CD73, CD90 and CD105 of the cultured cells. Over 94% of cells expressed these markers, indicating that the majority of them were MSCs.

### MSC-EVs isolation, labeling and identification

Ultrahigh-speed differential centrifugation was used to collect EVs. Briefly, MSCs at 50–60% confluence were dissociated into single cells and transferred into Eppendorf (EP) tubes and centrifuged at 300 × g for 10 min at 4 °C. A second centrifugation with 2,000 × g for 10 min at 4 °C was applied to remove the cell fragments. The supernatant was centrifuged a third time with 10,000 × g at 4 °C for 30 min to further remove organelles. Finally, the supernatant was centrifuged again with 120,000 × g at 4 °C for 90 min in an ultracentrifuge using a SW32Ti rotor (Beckman Coulter, USA) to obtain the EVs pellet from the bottom, which were then dissolved in NS and stored at − 80 °C for further experiments.

To label the EVs, PKH26 (1:200, Sigma) or Dio (1:200, Thermo) was added into the supernatant after the final centrifugation and incubated for 2 min. Then, centrifugation with 120,000 × g was performed again at 4 °C for 90 min. EVs combined with PKH26 or Dio were collected from the bottom of the centrifugal tube. Labeled EVs were washed with NS or PBS and centrifuged with 10,000 × g at 4 °C for 90 min. PKH26 - or Dio-labeled EVs were then able to be traced by red or green fluorescence under confocal microscopy (Zeiss, LSM900, Germany).

To quantify the particle number of MSC-EVs, an EVs ELISA complete kit (CD81 detection, System Biosciences) was applied according to the manufacturer's instructions. Zetasizer (Malvern Company, Nano series-Nano-ZS, England) was used to analyze the size distribution of the EVs. The shape of the EVs was observed with transmission electron microscopy (TEM). WB was applied to identify the expression of CD9, CD63, Hsp70 and ALIX protein of MSC-EVs.

For transmission electron microscopy (TEM), collected EVs’ pellets were fixed in a solution of 2% paraformaldehyde, 2% glutaraldehyde and 0.05 M phosphate buffer. Then, EVs were loaded onto copper grids, contrasted using 2% uranyl acetate, dried and observed using a TECNAI T12 TEM (FEI, USA).

### Co-culture of photoreceptor with microglia cell

BV2 cells, a mouse microglial cell line, were obtained from Qinqi (Shanghai, China). 661 W cells, a mouse photoreceptor-like cell line, were a gift from Prof. Jing Zhuang (The Ophthalmological Center of Sun Yat-Sen Hospital, Guangzhou, China). High-glucose Dulbecco’s modified Eagle medium was applied to culture both BV2 cells and 661 W cells. BV2 cells were firstly cultured in a transwell chamber (Millipore, USA) for 6 h and then treated with lipopolysaccharide (LPS, 10 µg/mL, Sigma) for 24 h to be induced into active state. Then, LPS was washed out and BV2 was moved into the co-culture system, where 661 W cells were seeded and had been cultured for 24 h. Then, 661 W cells were co-cultured with the activated BV2 cells for 48 h, with MSC-EVs or NS, and then, both cell lines were either fixed or collected for further experiments.

### TUNEL staining

All TUNEL-staining procedures were performed using an In Situ Cell Death Detection Kit, POD (Roche Applied Science, 11,684,817,910, USA) under the instructions of the manufacturer. DAPI (4′,6-diamidino-2-phenylindole; 1:1000, Invitrogen) staining was used to observe the nuclei of 661 W. All slides were then observed under a microscope (Zeiss, Germany).

### Treatment and exosome tracing

Morphological changes in the rd10 photoreceptors start as early as P10, indicated by the shortening of outer segments of rods and cones [3, 19]. We subsequently injected the littermates of rd10 mice with MSC-EVs or NS at P14 (when the eye opens) and again at P21 (after the onset of rod degeneration). Either 1 μl MSC-EVs (1 × 10^10^ in 1 μl) or NS was intravenously injected into both eyes of the animal [20]. At P27, the visual behavior of animals was tested with an optomotor system; mice were then dark-adapted overnight, and the retinal light responses were examined by electroretinogram (ERG). Animals were then killed, and retinas were collected for either western blotting or immunostaining. The experimental methods and protocol are shown in Additional file 1: Fig. S1.

### Optokinetic response test (optomotor)

To evaluate the visual behavior, the optomotor test was carried out on mice at P27 after overnight dark adaptation. Briefly, a mouse was placed on a platform surrounded by four computer screens which presented moving vertical sine gratings driven by a program written with MATLAB software. The gratings with increasing spatial frequencies (including 0.1, 0.2, 0.25, 0.3, 0.35, 0.4, 0.5 and 0.6 cycles/degree, 100% contrast) were displayed for 30 s in both clockwise and counterclockwise directions. For each frequency, head movements of the mouse were recorded with a video camera, and a head rotation following the direction of the moving grating was considered a positive optokinetic response. The mean luminance of the monitor screens was 30 μw/cm^2^. The highest spatial frequency at which an optokinetic response was observed was recorded as the visual acuity of the responsive eye.

### Electroretinogram

To measure the retinal light responses, the electroretinogram (ERG) test was carried out as previously described by the RETI scan system (Roland Consult, Wiesbaden, Germany) [21]. Briefly, mice were anesthetized by injecting 1.25% tribromoethanol (0.20 mL/10 g body weight) intraperitoneally. After mydriasis with atropine sulfate (0.5%), the mice were placed on a heating platform maintained at 37 °C. Gold-plated wire loop electrodes were placed on the corneal surface serving as the active electrode. Stainless steel needle electrodes were inserted in the skin near the eye and tail, serving as the reference and ground electrodes respectively. Mice were presented with full-field green-light flashes at intensities of 0.01, 0.03, 0.1, 0.3, 1.0 and 3.0 cd •s/m^2^. They were then light-adapted for 5 min under a bright green background (20 cd • s/m^2^), and then, the photopic responses to green flashes of 10 cd • s/m^2^ were recorded. The a-wave and b-wave amplitudes and the time to a-wave and b-wave peaks were analyzed with the RETI-scan software. For each animal, data were obtained from the most responsive eye.

### Tissue processing

The eyes were removed quickly after overdose anesthetic and fixed in 4% paraformaldehyde (PFA) for 30 min at 25 °C (room temperature). For whole-mount staining, the retinas removed from the eyes were cut into a clover shape under a stereomicroscope. The retinas were moved into 0.01 M PBS (phosphate-buffered saline) in 24-well plates at 4 °C for further experiments. For cryo-sectioning, only the cornea and lens were removed, and the eye cups were osmotically dehydrated at 4 °C in 0.01 M PBS containing 35% sucrose for at least 24 h. Then, samples were embedded in OCT (optimal cutting temperature compound, Tissue Tek, Torrance, USA) and quickly moved into − 80 °C freezer. The retinas were cryo-sectioned lengthwise at a thickness of 10 μm by using frozen slicer (Thermo). Sections that contained the optic disk (OD) were mounted on glass slides for further experiments.

### Immunocytochemistry

For whole-mount staining, the retinas were placed into 24-well plates with 0.5% PBS-Triton X-100 (PBST), washed three times and then incubated in 0.5% PBST containing 3% normal donkey serum (NDS) and 1% bovine serum albumin (BSA) for 1 h. Primary antibodies were added into the 24-well plates, which were processed for 48 h at 4 °C on a shaker. For cryo-sections, slices were washed three times with 0.3% PBST and then incubated in 0.3% PBST containing 3% NDS and 1% BSA for 1 h at 25 °C. Primary antibodies were added to cover the retinal tissues and incubated for 12 h at 4 °C. Then, tissues were washed and incubated with secondary antibodies for 2 h at room temperature.

The primary antibodies used included rabbit anti-Iba1 (1:1000, Wako, 019–19,741), rabbit anti-GFAP (1:2000, Abcam, ab7260), mouse anti-rhodopsin (1:1000, Millipore, MAB5356), rabbit anti-opsin, Red/Green (1:1000, Millipore, #AB5405), mouse anti-PSD95 (1:500, CST, #36,233), rabbit anti-recoverin (1:1000, Millipore, #AB5585), mouse anti-GS (1:1000, MAB302), mouse anti-F4/80 (1:200, AbD Serotec, MCA497), rabbit anti-CD206 (1:500, CST, #24,595) and rat anti-CD68 (1:500, GTX41865, Genetex). The secondary antibodies included donkey anti-mouse or donkey anti-rabbit (conjugated to Alexa-488 or 594, 1:1000, Invitrogen).

### Western blotting

Under a stereomicroscope, the retinas were quickly removed from the eyecups and then put into icy lysis buffer, and lysed for 30 min at 4 °C. The concentration of the lysed retinal proteins was detected using a bicinchoninic acid (BCA) assay kit (RiboBio, China), and at least 30 μg of retinal protein was loaded per lane and separated electrophoretically on a 10% (v/v) SDS–polyacrylamide gel and then transferred to a polyvinylidene difluoride (PVDF) membrane (Millipore, USA). PVDF membranes were blocked with 5% (w/v) skimmed milk and 0.1% Tween-20 in Tris-buffered saline for 90 min and incubated in primary antibodies on a shaker overnight at 4 °C. Then, the PVDF membranes were hatched with secondary antibodies, donkey anti-mouse or donkey anti-rabbit (CST, USA), on a shaker for 2 h at 25 °C. The PVDF membranes were washed three times, and the results were visualized using a chemiluminescence system (Bio-Rad). The semi-quantification of the blots was performed using ImageJ software.

The primary antibodies used included mouse anti-IL-1β (1:1000, CST, #12,242), mouse anti-IκBα (1:1000, CST, #4814), rabbit anti-Phospho-IκBα (Ser32) (1:1000, CST, #5209), mouse anti-NF-κB p65 (1:1000, CST, #8242), rabbit anti-CD9(1:1000, CST, #13403S), rabbit anti-CD63 (1:1000, CST, #55051S), rabbit anti-Hsp70 (1:1000, CST, #4873S) and rabbit anti-ALIX (1:1000, CST, #92880S). The secondary antibodies were anti-rabbit-HRP (1:4000, CST) and anti-mouse-HRP (1:4000, CST).

### RNA-seq and real-time RT-PCR

To check the gene expression, RNA was extracted from mouse retina and the cDNA, DNA and small RNA libraries were sequenced on the Illumina sequencing platform by Genedenovo Biotechnology Co., Ltd. (Guangzhou, China). For miRNA-seq, we collected MSC-Exos as previously described, and samples were sequenced on the Illumina sequencing platform by RiboBio Biotechnology Co., Ltd. (Guangzhou, China). For RT-PCR, retinal tissues were harvested immediately using TRIzol reagent (Takara), and total RNAs were extracted using phenol/chloroform, from which 1 mg of RNA was reverse transcribed using the Superscript cDNA kit (Takara). The resulting cDNA was used as a template for subsequent PCR amplification with specific primers (Gene Company Limited). The relative mRNA expression levels of the target gene were normalized to the expression levels of GAPDH determined using the 2^−ΔΔCT^ method. An All-in-One™ miRNA qRT-PCR Detection Kit 2.0 was used to detect the expression of miR-146a-5p. The expression of miR-146a-5p levels was normalized to the expression levels of U6. The qRT-PCR primers were purchased from Guangzhou GeneCopoeia (Guangzhou, China, http://www.igenebio.com/) and are listed in Additional file 2: Table S1.

### The dual-luciferase experiment

To determine whether miR-146a directly combined with downstream target Nr4a3, dual-luciferase experiment was carried out. Four plasmids were constructed and overexpressed in 293 T cells. These included the seed sequence of miR-146a-5p, the Nr4a3 mRNA 3′ UTR, the mutant-1 Nr4a3 mRNA 3′ UTR and the mutant-2 Nr4a3 mRNA 3′ UTR. We acquired the target gene sequence by the following primers: mmu-miR-146a-5p seed sequence (F-5’TGTGGAAAGGACGCGGGATCTACAGGGCTGGCAGGATCTG3’, R-5’ TCACCATGGTGGCGACCGGGCTGACACTCAACTGAGCA 3’), Nr4a3 mRNA 3′ UTR (F-5’GATCGCCGTGTAATTCTAGATTTTCCATTCATGATCATGGTAGC3’, R-5’CCGGCCGCCCCGACTCTAGACGAATGAGCCATGGGGAAGGAAATC3’), the mutant-1 Nr4a3 mRNA 3′ UTR (F-5’GATCGCCGTGTAATTCTAGATTTTCCATTCATGATCATGGTAGC3’, R-5’CCGGCCGCCCCGACTCTAGACGAATGAGCCATGGGGAAGGAAATC3’), the mutant-2 Nr4a3 mRNA 3′ UTR (F-5’GATCGCCGTGTAATTCTAGATTTTCCATTCATGATCATGGTAGC3’, R-5’CCGGCCGCCCCGACTCTAGACGAATGAGCCATGGGGAAGGAAATC3’). Reporter constructs were confirmed by sequencing before used. Luciferase activity was measured 48 h after transfection of 293 T cells with one of these four plasmids using Dual-Luciferase® Reporter Assay System (Promega, #E1910). Coexpressed Renilla luciferase on the pmirGLO vector was used as an internal control to normalize the firefly luciferase activity. We also added a positive control group, which was miR-146b and its known target gene TRAF6 [22].

### Analysis of fluorescence intensity and gray value

ImageJ software was used to analyzed the fluorescence intensity in a gray scale. To quantify the fluorescent intensity, integrated gray levels over full sections were performed for Iba1, GFAP and recoverin staining, while for PSD95, the gray level over the stained region (limited to the threshold) was measured by ImageJ. For F4/80, CD63 and CD206 staining, ImageJ was used to count the fluorescence-positive cells.

### Statistical analysis

All data are expressed as the mean ± SEM. ANOVA and Student’s *t* test were performed using GraphPad Prism 5. Statistical significance was set at *P* < 0.05, and *P* values < 0.01 were considered highly significant. The total numbers of animals or retinas are indicated by “n” in each group.

## Results

### Characterization of MSC-EVs extracted from umbilical cord MSCs

**Fig. 1:**
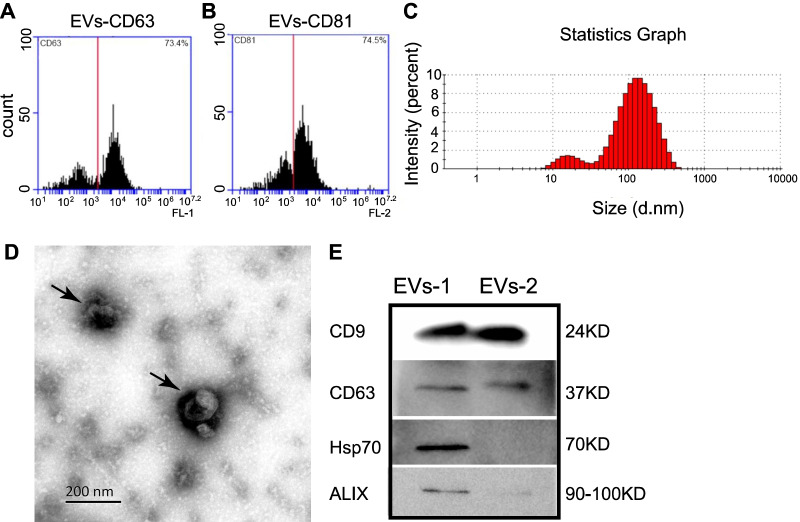
Characterization of MSC-EVs. **A**–**B** Flow cytometry results showed 73.4% and 74.5% of EVs were positive for CD63 A and CD81 (B). **C** Particle size analysis of the EVs showing the distribution of EVs with different sizes. Around 75% of the particles had the size of exosomes, i.e., 30–200 nm. **D** Morphologies of two typical EVs taken by transmission electron microscopy (indicated by arrows). E. Western blotting showing the expression of EVs’ markers (CD9, CD63, Hsp70 and ALIX) in two batches of exosomes, EVs-1 and EVs-2, with lower concentration

MSCs were successfully extracted from explants of human umbilical cords and subcultured. Cells were then identified (as we described previously [23, 24]) by positive staining for CD44, CD73, CD90 and CD105, and negative for CD34 and CD45, which was further confirmed by flow cytometry (data not shown). EVs were then enriched by differential ultra-high-speed centrifugation and flow cytometry and were identified by their expression of the membrane surface markers CD63 and CD81 (*n* = 3, Fig. 1A and B). There were 73.4% CD63-positive and 74.5% CD81-positive components, indicating that a high number of EVs were present in the MSCs (Fig. 1A and B). Particle size analyses showed that the EVs with the size of exosomes, i.e., 30–200 nm EVs, occupied 74.7 ± 3.3% of the total EVs [25], indicating the enrichment of exosomes (*n* = 3, Fig. 1C). The next two enriched groups were EVs with a size of 200–1000 nm (14.0 ± 1.3%) followed by those with a size of 10–30 nm (11.1 ± 1.3%). TEM further confirmed that the enriched particles exhibited the typical morphology of exosomes, which were cup-shaped with a double-membrane structure (*n* = 3, Fig. 1D). Expression of the EVs’ markers, including CD9, CD63, Hsp70 and ALIX, was further confirmed by western blotting (WB) with examples from two batches of EVs (from different batches with lower concentration of EVs-2 therefore lower expression of Hsp70 and ALIX than EVs-1) [26] (Fig. 1E). Taken together, our results demonstrate that we have successfully collected MSC-EVs.

### Uptake of MSC-EVs in retina

In order to assess the possible neuroprotective effect of MSC-EVs, we examined whether MSC-EVs could be endocytosed by retinal cells, including neurons and glial cells. PKH26- or Dio-labeled EVs were injected intravitreally into WT mice and whole-mount retinas were extracted after 3 h, 6 h, 12 h and 24 h after the injection. At 3 h, a low uptake of MSC-EVs was observed in the retinal interstitium (*n* = 3, Additional file 3: Fig. S2A). After 6 h, MSC-EVs (red dots) was found inside retinal cells and the number gradually increased (*n* = 3, Additional file 3: Fig. S2B–D). Studies have shown that MSC-EVs remained in the vitreous humor for up to 4 weeks after injection [27]; therefore, the effects of MSC-EVs may persist during our whole experimental procedure (2 weeks).

Next, we assessed the types of retinal cells that took up MSC-EVs. At 24 h after injection, MSC-EVs diffused into every retinal layer (*n* = 3, Additional file 4: Fig. S3A–C) including ONL, INL and GCL, indicating that almost all types of retinal cells could endocytose. Immunofluorescence results on whole-mount retina showed that almost no MSC-EVs were endocytosed by astrocytes (purple arrows, Additional file 4: Fig. S3D), but more by retinal microglia/macrophage (stained with Iba1, Additional file 4: Fig. S3F) and Müller glia (stained with GS, Additional file 4: Fig. S3E). Similar results were also found in rd10 mice, with PKH26- and Dio-labeled EVs found in ONL and INL. (*n* = 3, Additional file 5: Fig. S4A–B).

### MSC-EVs promote visual functions in rd10 mice

**Fig. 2:**
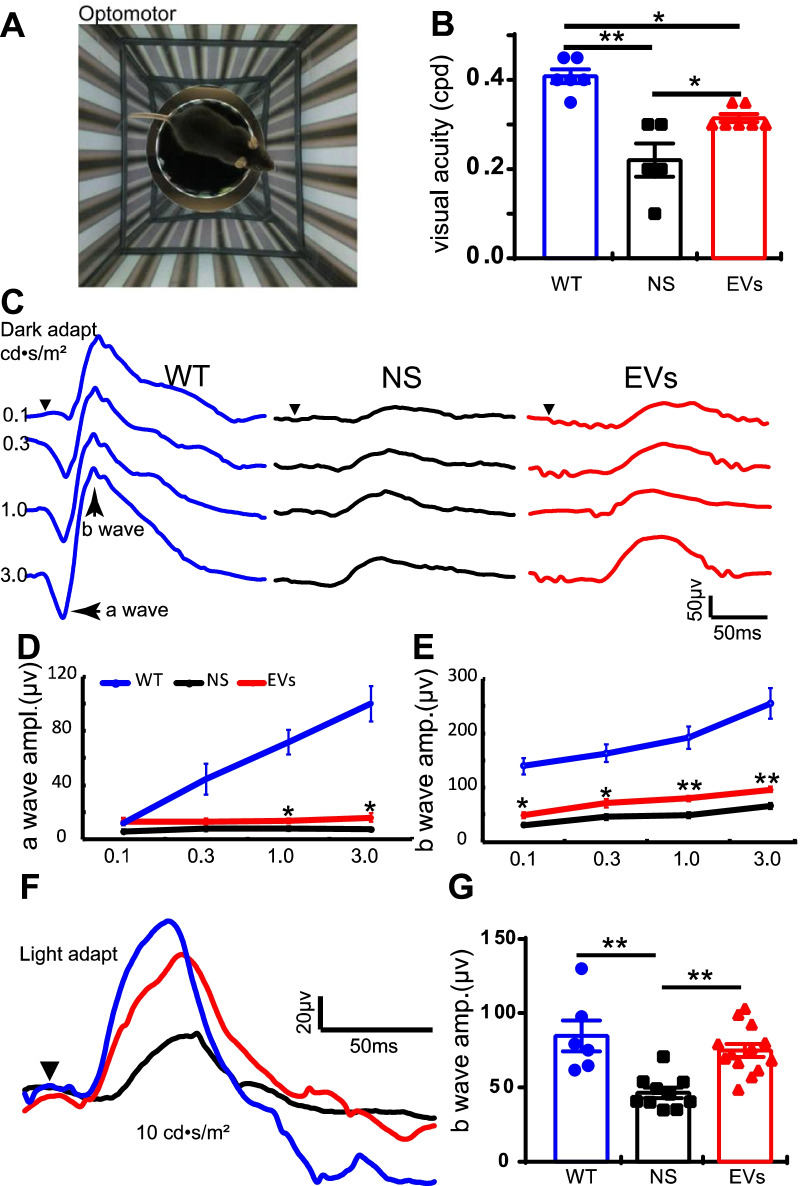
MSC-EVs improves visual function in rd10 mice. **A** Illustration of the optomotor system. **B** Quantification of the visual acuity in WT, NS-treated and MSC-EVs-treated rd10 mice. **C** Representative ERG traces under various light intensities ranging from 0.1 to 3.0 cd • s/m^2^ under dark adaptation in WT (blue), NS-treated rd10 mice (black) and MSC-EVs-treated rd10 mice (red) at P28. Arrowhead shows the starting time point of the flash. **D**, **E** Average peak amplitudes of dark-adapted a-wave (D) and b-wave (E). **F** Representative ERG traces at 10.0 cd • s/m^2^ flash under light adaptation with 20 cd • s/m^2^ background. **G** Average b-wave amplitudes of light-adapted condition in three groups. Data are represented as mean ± SEM. Student’s t test and one-way ANOVA test were applied. * *P* < 0.05, **, *P* < 0.01

To assess the protective effects of MSC-EVs, we first evaluated the visual behavior by the optomotor response at P27 (Fig. 2A), and the retinal light responses the next day. In WT mice, the visual acuity was 0.41 ± 0.02 cycles per degree (cpd) (*n* = 6, Fig. 2B), while it significantly decreased to 0.22 ± 0.04 cpd in NS-treated rd10 mice (*n* = 5, Fig. 2B, *p* = 0.001). MSC-EVs treatment significantly increased the visual acuity of rd10 mice to 0.31 ± 0.01 cpd (*n* = 7, *P* = 0.016 vs. NS group, Fig. 2B).

Next, we evaluated the neuronal function of the photoreceptors at P28 using ERG. ERG is a noninvasive electrophysiological technique that records the ability of neurons to respond to a light flash, with a-wave and b-wave reflecting the group responses of photoreceptors and their downstream neurons, the bipolar cells. In WT mice, ERG traces showed strong retinal light responses under both dark-adapted (blue line, Fig. 2C) and light-adapted conditions (Fig. 2F). The light responses greatly decreased in rd10 mice (black lines, Fig. 2C,D and E) under all flash intensities tested, and MSC-EVs treatment significantly rescued the light responses of rd10 retinas (red lines, Fig. 2C,D and E). Statistically, MSC-EVs significantly improved the a-wave amplitude at strong flash intensities (> 1.0 cd • s/m^2^, Fig. 2D) and the b-wave amplitude (Fig. 2E and G) at all flash intensities. While the time to a-wave or b-wave peak was extended in rd10 mice, MSC-EVs hardly helped to shorten them (data not shown). Similarly, under light adaptation, a significant improvement of the b-wave amplitude was observed after MSC-EVs treatment in rd10 mice. No difference in the time to b-wave peak was observed. Altogether, our results showed that MSC-EVs improved the visual function in rd10 mice.

### MSC-EVs improve the survival and structure of the photoreceptors in the rd10 retina

**Fig. 3:**
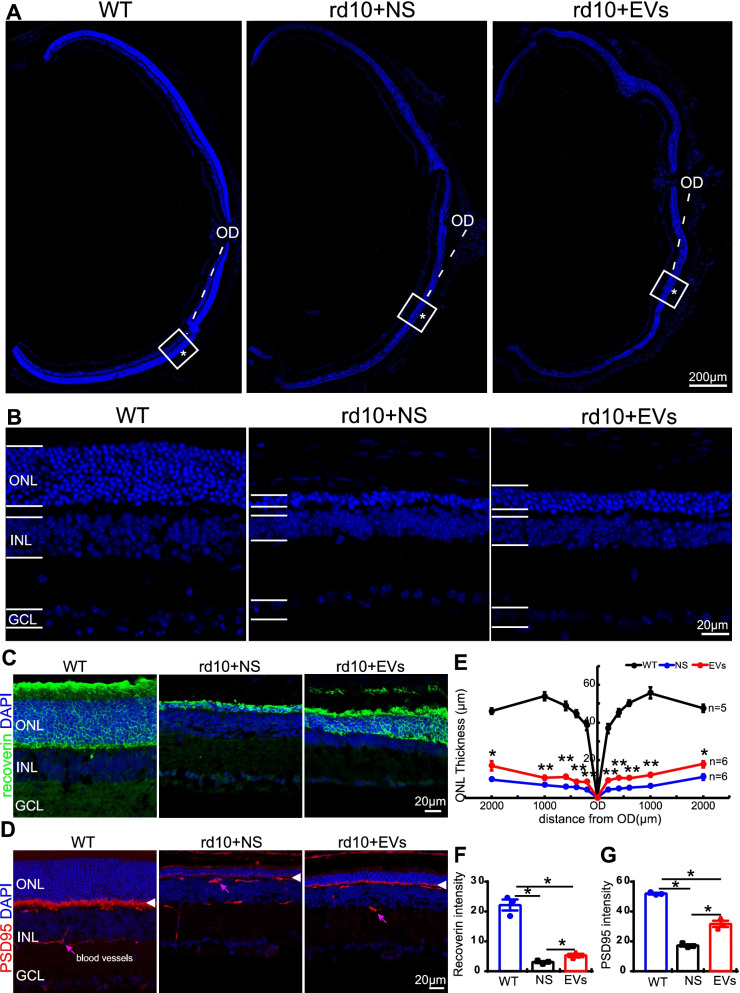
MSC-EVs promote photoreceptor survival in rd10 mice at P28. **A** Images of eye cups with retinas stained with DAPI from three animal groups, with squared regions at 600 µm away from the center of the optic disk (OD) enlarged in **B**. ONL layer where photoreceptor somas are located (*) became thinner in rd10 mice, while MSC-EVs increased its thickness. **C**, **D** Images of immunofluorescence staining with an antibody against recoverin (green, C) or PSD95 (red, D) and DAPI (blue) of retinal slices in three animal groups. Arrowheads in D point to the photoreceptors presynaptic terminals that are labeled by anti-PSD95. Pink arrows point to blood vessels. **E** Quantification of the ONL thickness at 200 µm, 400 µm, 600 µm, 1000 µm and 2000 µm away from the OD at P28. **F** Quantification of the mean gray intensity in the three animal groups of recoverin (F) and PSD95 (**G**) in three animal groups. OD, optic disk; ONL, outer nuclear layer; INL, inner nuclear layer; and GCL, ganglion cell layer. Data are shown as mean ± SEM. * *P* < 0.05, **, *P* < 0.01. Student’s t test and one-way ANOVA were applied

We next assessed the protective effect of MSC-EVs on photoreceptors survival rate at P28, the end of the fast degeneration phase [1, 16]. Given that nearly 97% of photoreceptors are rods and their somas are located in the outer nuclear layer (ONL) of the mouse retina [28], we evaluated the photoreceptors survival rate using the ONL thickness as an index. Examples of cryo-sections of eye cups from three groups are shown in Fig. 3A, and regions at 600 µm from the optic disk were enlarged and are shown in Fig. 3B. As rd10 retina degenerates from center to periphery, we measured the ONL thickness at 200 µm, 400 µm, 600 µm, 1000 µm and 2000 µm away from the center of OD. The ONL thickness in the NS-treated rd10 retina was greatly reduced (blue line) compared with WT (black line), and MSC-EVs treatment (red line) greatly improved it (Fig. 3E). For example, at 600 µm away from the center of OD, the ONL thickness of NS-treated group was 6.5 ± 0.3 µm (11% of WT, *P* = 0.001 vs. WT, *n* = 6, Fig. 3E, blue) and MSC-EVs-treatment increased it to 12.0 ± 0.8 mm (21.6% of WT, *n* = 6, Fig. 3E, *P* = 0.001 vs. NS). Therefore, MSC-EVs treatment alleviated photoreceptor death in rd10 mice.

The structure of photoreceptors was further explored by staining retinas for recoverin and PSD95. Recoverin is a 23-kDa calcium-binding protein present in photoreceptors, which functions as a calcium sensor that regulates rhodopsin phosphorylation through inhibition of rhodopsin kinase (Fig. 3C, green). We analyzed the mean gray intensity of recoverin per 320 µm × 320 µm (per section) in WT, NS-treated and MSC-EVs-treated rd10 mice. In WT mice, recoverin stained heavily the somas and the outer segments (OS) as well as the inner segments (IS) of photoreceptors. As photoreceptors degenerated in rd10 mice, outer segments became shorter and recoverin staining became weaker; in MSC-EVs-treated mice, the staining intensity was higher and the outer segments were longer and more numerous. Statistically, the mean gray intensity of recoverin in NS-treated rd10 mice was significantly reduced from 22.2 ± 1.9 in WT (*n* = 3) to 3.1 ± 0.3 (*n* = 3, *p* < 0.001, Fig. 3C and F). After MSC-EVs treatment in rd10 mice, the mean gray intensity of recoverin was significantly increased to 5.3 ± 0.5 (*n* = 3, *p* = 0.023, Fig. 3C and F). The length of OS and IS was recovered from 3.4 ± 0.5 µm in NS-treated group (*n* = 3) to 7.1 ± 0.7 µm (*n* = 3, *p* = 0.01 vs. NS-treated group, data not shown). Thus, MSC-EVs treatment slowed down rod degeneration in rd10 mice.

PSD95 is a postsynaptic-associated protein which, in retina, is present in the presynaptic terminals of photoreceptor terminals. In WT mice, a strong expression of PSD95 was observed as a thick band in OPL, and the staining became much weaker and lost the band structure in rd10 retina. MSC-EVs treatment restored the band structure of PSD95 in OPL and the staining became stronger. Statistically, the mean gray intensity of PSD95 was decreased from 52.0 ± 0.5 in WT to 17.3 ± 0.8 in NS-treated rd10 mice (*n* = 3, *p* < 0.001 vs. WT), and MSC-EVs restored the intensity to 31.7 ± 2.1 (*n* = 3, *p* = 0.028 vs. NS, Fig. 3D and G). These findings suggest that MSC-EVs preserved photoreceptor structures in the rd10 retina.

### MSC-EVs inhibit reactive gliosis and immunoproliferation in rd10 retina

In retina, reactive gliosis is the indicator of inflammation, which is characterized by the upregulation of GFAP in Müller glial cells and the activation of microglial/ macrophages. As reactive gliosis in rd10 mice appears as early as P10, we assessed whether MSC-EVs repress the activation of microglia/macrophage and Müller glial cells in the rd10 retina by immunostaining for Iba1 (microglia/macrophage), F4/80 (M0 macrophage), CD63 (M1 macrophage), CD206 (M2 macrophage) and GFAP (Müller glial).

**Fig. 4:**
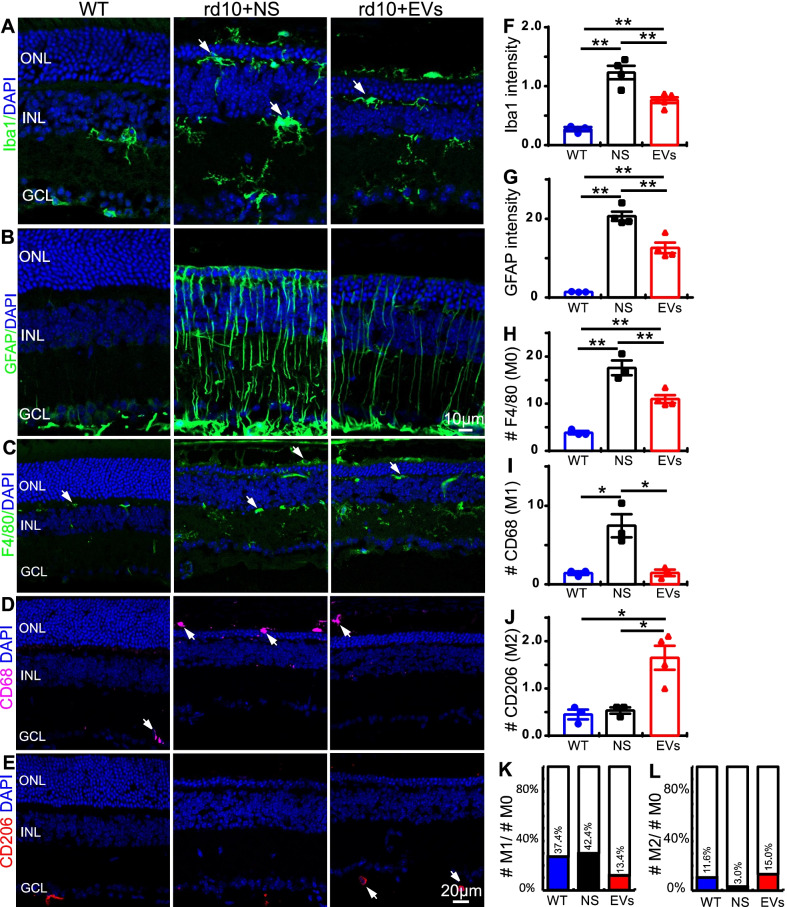
MSC-EVs inhibit the reactive gliosis and immunoproliferation in the rd10 retina. **A** Retinal sections stained with DAPI (blue) and for Iba1 (green) in WT, NS-treated rd10 and MSC-EVs-treated rd10. In the rd10 retina, microglial cells presented as active state and distributed throughout the whole retina. MSC-EVs decreased its expression. **B** Retinal sections stained for GFAP (green). In the rd10 retina, GFAP was strongly expressed in Müller cells and extended from GCL to retinal OS; MSC-EVs decreased its expression. **C**-**E** Retinal sections stained for F4/80 (green, C), CD68 (pink, D) or CD206 (red, E). **F**, **G** Quantification of the Iba1-positive fluorescence intensity (F) and GFAP-positive fluorescence intensity (G). **H**-**J** Quantification of the number of F4/80 (M0 macrophage) (H)-, CD68 (M1 macrophage) (I)- and CD206 (M2 macrophage) (J)-positive cells. **K**, **L** Quantification of the ratio of M1/M0 number (K) and M2/M0 number (L). The MSC-EVs treatment reduced the fluorescence intensity of Iba1, the GFAP neurite intensity and the number of M0 and M1 macrophages in the rd10 retinas. White arrow indicated the cell somas of positive fluorescence. Data are shown as mean ± SEM. * *P* < 0.05, **, *P* < 0.01. One-way ANOVA

The active state of microglial cell was examined by Iba1 staining. Iba1 specifically labels the somas and neurites of microglial cells. In the WT retina, Iba1-stained microglia cells were in a resting state with long branches and small somas and were mainly distributed in the inner retinal layer [3]. No microglial/macrophage was detected in the ONL of WT mouse retinas (Fig. 4A). In the NS-treated rd10 retina at P28, microglial/macrophage was found across all layers of the retina, especially in the ONL and outer segments, and microglial cells showed reactive morphologies (changed shapes with large somas and retracted branches) (Fig. 4A). MSC-EVs treatment significantly reduced the mean Iba1-positive fluorescence intensity (Fig. 4F). The average intensity of Iba1-positive fluorescence in NS-treated rd10 retina was 1.2 ± 0.1 per image (*n* = 4, with size of 320 µm × 320 µm), which was significantly reduced to 0.8 ± 0.1 per image after MSC-EVs treatment (*p* = 0.001 vs. NS, *n* = 5, Fig. 4 F).

We then assessed the expression of GFAP in the WT and rd10 retinas with immunostaining. As previously reported [21, 29], in normal retina, GFAP was mainly expressed in astrocytes located in the ganglion cell layer (GCL) (Fig. 4B). In the NS-treated rd10 group, the GFAP staining extended along the processes of Müller glia across the entire retina (Fig. 4B). Treatment with MSC-EVs decreased the expression of GFAP in Müller cells. The intensity of GFAP-positive fluorescence was quantified: MSC-EVs significantly decreased the intensity from 20.7 ± 1.2 per image (size of 320 µm × 320 µm) in NS group to 12.6 ± 1.3 per image (*P* = 0.007, *n* = 4, Fig. 4G).

In addition, we assessed the activation level of macrophages in the WT and rd10 retinas by staining for F4/80, CD68 and CD206. At a resting state, macrophages present as M0 type which could be labeled by F4/80 in mouse. Under inflammatory conditions, these macrophages are transformed either into pro-inflammatory M1 type, which express CD68, or into anti-inflammatory M2 type, which express CD206. In the WT retina, only few F4/80-positive cells were observed in whole retina, while many M0-type macrophages were present in ONL and INL of NS-treated rd10 mouse retinas (Fig. 4C). Treatment with MSC-EVs decreased the number of M0 type macrophages. Statistically, MSC-EVs significantly decreased the number of M0 type macrophage from 17.6 ± 1.6 per image (size of 320 µm × 320 µm) in NS group to 11.0 ± 0.8 per image (*P* = 0.009, Fig. 4H). Similar results were observed with CD68 staining (Fig. 4D), and MSC-EVs significantly decreased the number from 7.5 ± 1.5 per image in NS group to 1.5 ± 0.4 per image (*P* = 0.016, Fig. 4I). The number of CD206-positive cells was also quantified, and MSC-EVs significantly increased the number from 0.5 ± 0.1 per image in NS group to 1.7 ± 0.3 per image (*P* = 0.015, Fig. 4E and J). We also quantified the M1/M0 and M2/M0 ratios, and these results are presented in Fig. 4K, L. These observations indicate that MSC-EVs inhibit reactive gliosis and immunoproliferation.

### MSC-EVs regulate the gene expression of inflammatory cytokines in the rd10 retina

**Fig. 5:**
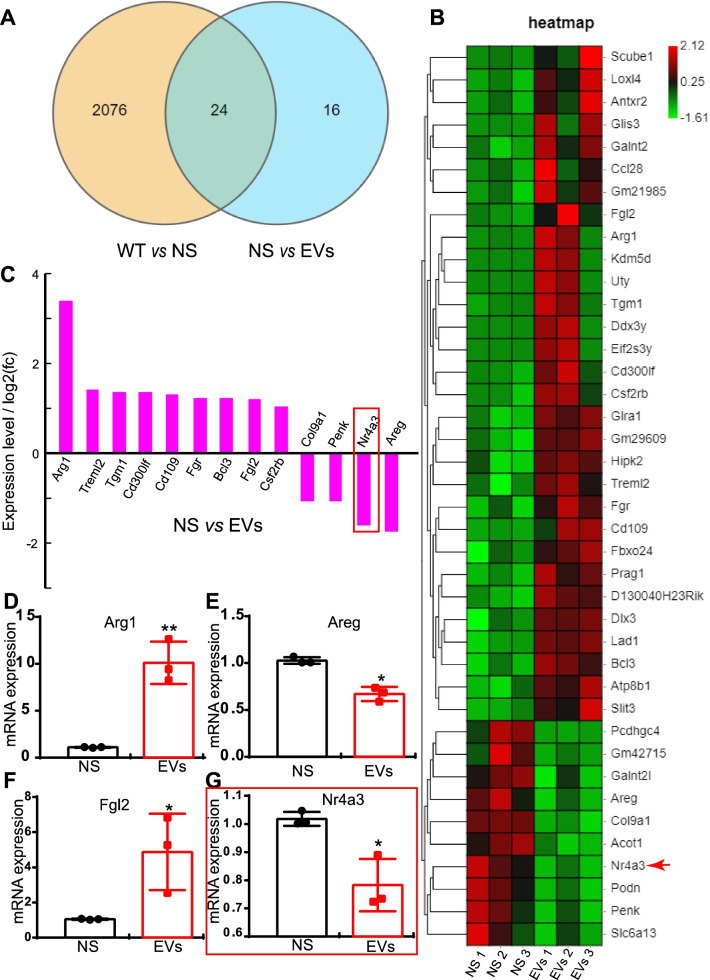
MSC-EVs regulate the gene expression of inflammatory cytokines in the rd10 retina. **A** A pie chart of RNA-seq results in WT, NS-treated and MSC-EVs-treated rd10 retinas showing the number of genes whose expression differs between the groups. **B** Heat map of RNA-seq results showing the 40 differentially expressed genes between NS-treated and MSC-EVs-treated rd10 retinas. **C** The expression level of 13 differentially expressed genes between NS-treated and MSC-EVs-treated rd10 retinas which are related to immunity and inflammation. **D**-**G** qPCR quantification of 4 differentially expressed genes between NS-treated rd10 and MSC-EVs-treated rd10 retinas. Data are represented as mean ± SEM. A Student’s t test was applied for qPCR data

As MSC-EVs inhibit the inflammation in rd10 retina, we next assess whether MSC-EVs modulate the transcriptome of genes related to immunity and inflammation by RNA-seq. We found that over 2000 genes were differentially expressed between normal and rd10 retina and 40 genes differentially expressed between NS-treated and MSC-EVs-treated rd10 mice (Fig. 5A). The RNA-seq data have been uploaded to NCBI now (https://www.ncbi.nlm.nih.gov/geo/query/acc.cgi?acc=GSE200621). The 40 genes are further illustrated in a heat map (Fig. 5B). Among them, 13 were related to immunity and inflammation, with 9 upregulated and 4 downregulated by MSC-EVs (*n* = 3, Fig. 5C). We used qPCR to further verify the RNA-seq findings with examples illustrated in Fig. 5D-G. MSC-EVs significantly increased the mRNA level of the anti-inflammatory cytokine, Arg1 (arginase 1) [24] (Fig. 5D) and decreased that of the pro-inflammatory cytokine Areg [30] (Fig. 5E). Fgl2 (fibrinogen-like 2), a negative regulator of macrophages, was also significantly increased by MSC-EVs (Fig. 5F), while Nr4a3 (Nuclear receptor subfamily 4, group A, member 3), a transcription factor that activates NF-κB [31], was significantly decreased (Fig. 5G). Thus, MSC-EVs promote the expression of anti-inflammatory cytokines and suppress the expression of pro-inflammatory cytokines in rd10 retinas.

### MiRNAs are detected in MSC-EVs

After identifying several inflammatory cytokines that are modulated by MSC-EVs, we wondered whether MSC-EVs act through miRNAs that affect inflammatory pathways. To identify the therapeutic component of MSC-EVs, miRNA-seq was carried out on the MSC-EVs that we collected from human umbilical cord, and this revealed more than 20 miRNAs. The category and percentage of the top ten highly expressed miRNAs are displayed in Additional file 6: Fig. S5A, with the top three being miRNA-21-5p, miRNA-122-5p and miRNA-146a-5p. Many of these miRNAs are known to be related to immunity and inflammation and to affect inflammatory cytokines (such as IL-1β, Cox-2 and TNF-α) [9]. KEGG analyses revealed the 20 of the 119 signal pathways that are modulated by these 10 miRNAs, among them, TNF signaling pathway, NF-kappa-B (NF-κB) signaling pathway and toll-like receptors signaling pathway (Additional file 6: Fig. S5B). Thus, functional miRNAs are detected in MSC-EVs.

### MSC-EVs inhibit the activation of the NF-κB signaling pathway in rd10 retina

**Fig. 6:**
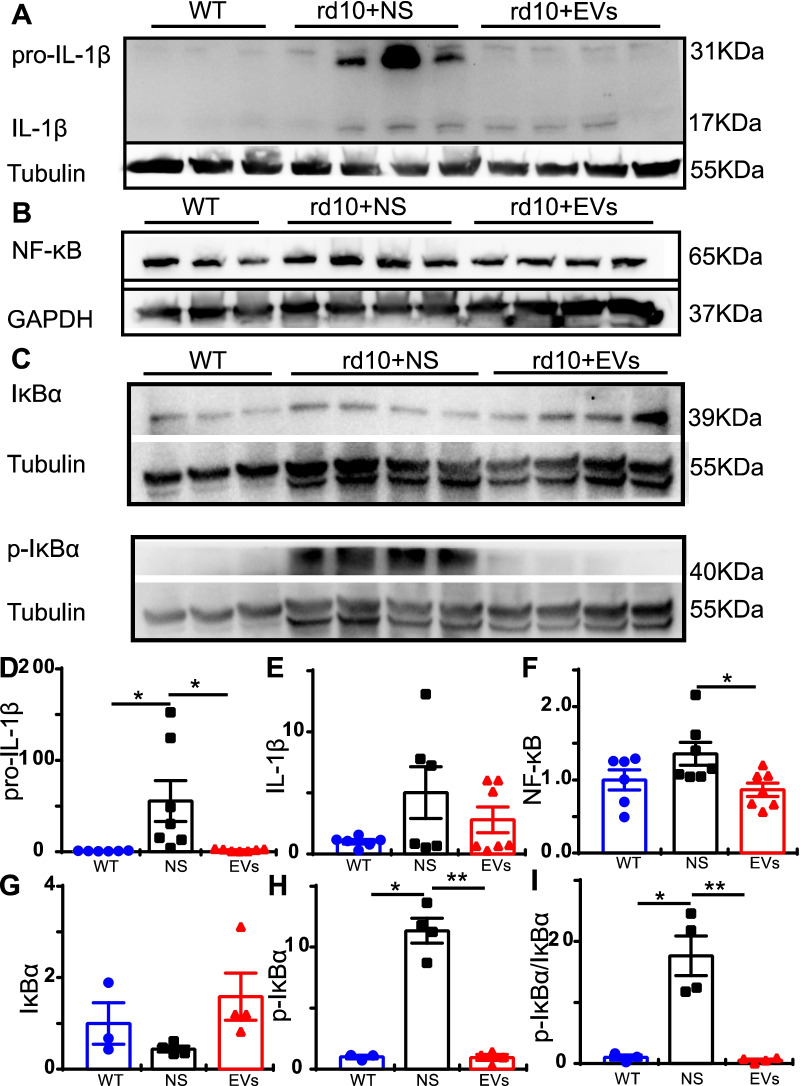
MSC-EVs inhibit the activation of NF-κB signaling pathway in rd10 mice. **A**-**C** Western blotting results of IL-1β and its precursor (A), NF-κB (B), IκBα and p-IκBα (C) protein. **D**-**I** Quantitative analyses of the expression levels of IL-1β precursor, IL-1β, NF-κB, IκBα, p-IκBα and p-IκBα/ IκBα normalized to that of WT retina. Data are represented as mean ± SEM. * *P* < 0.05, **, *P* < 0.01, one-way ANOVA

As KEGG analysis indicated that the NF-κB signaling pathway may be involved, we further assessed the protein levels of NF-κB signaling pathway including IL-1β, IκBα, p-IκBα and NF-κB proteins in retinal tissues by western blotting. Results showed that the expression of precursor IL-1β was significantly upregulated in the NS-treated rd10 retina relative to the WT retina and MSC-EVs decreased it significantly. The expression level of precursor IL-1β was decreased from 55.60-fold ± 22.3-fold of WT in NS-treated retinas to 2.09-fold ± 0.10-fold in the MSC-EVs-treated retinas (*P* = 0.03, Fig. 6A and D). A similar trend was observed also for Il-1β, though the changes were not significant (Fig. 6A and E). MSC-EVs also significantly decreased the expression of NF-κB/P65 (active form of NF-κB) from 1.36-fold ± 0.16-fold of WT in NS-treated rd10 retina (*n* = 7) to 0.87-fold ± 0.09-fold (*P* = 0.019, *n* = 7, Fig. 6B and F). The expression of IκBα, an inhibitor of NF-κB, and its phosphorylated form of p-IκBα were also examined. The expression of IκBα protein was similar across the three groups (Fig. 6C top panel), with no statistical difference (Fig. 6G). In contrast, the expression of p-IκBα greatly increased in rd10 retina, and MSC-EVs reduced its expression to near the normal level (Fig. 6C bottom panel). MSC-EVs significantly reduced the expression of p-IκBα protein from 11.35-fold ± 1.00-fold in NS group (*n* = 4) to 0.96-fold ± 0.25-fold (*P* < 0.001, *n* = 4, Fig. 6H). In addition, the ratio of p-IκBα protein to IκBα (p-IκBα/ IκBα) was also significantly decreased by MSC-EVs relative to the NS-treated rd10 group (*p* = 0.019, Fig. 6I). In summary, the NF-κB signaling pathway, which was activated in rd10 retina, became less active after MSC-EVs treatment.

### MSC-EVs reduce apoptosis and inflammation in vitro

**Fig. 7:**
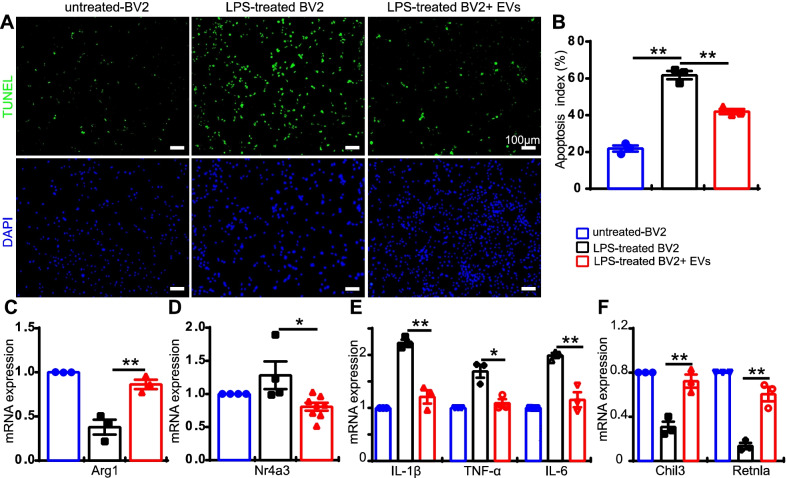
MSC-EVs suppress inflammation in vitro. **A** TUNEL staining of 661W cells co-cultured with inactive-BV2, LPS-activated-BV2 or LPS-activated BV2 + EVs. 661W cells were stained with DAPI (blue, bottom panel) and apoptotic cells were labeled with TUNEL staining (green, top panel). **B** The percentage of TUNEL positive 661W cells over DAPI positive cells in the three groups. C-H mRNA expression of Arg1 (**C**), Nr4a3 (**D**), IL-1β, TNF-α, IL-6 (**E**), Chil3 and Retnla (**F**) in the treated group relative to that of the untreated group. Data are represented as mean ± S.E.M. One-way ANOVA and Student’s t-test was applied for statistics

In order to explore the anti-inflammatory mechanism of MSC-EVs, a co-culture system consisting of microglial cell line BV2 with photoreceptor cell line 661 W was carried out. BV2 is a microglia cell line which expresses the microglia marker Iba1 (Additional file 7: Fig. S6), and 661 W is a photoreceptor-like cell line which expresses photoreceptor markers like recoverin and opsin (Additional file 8: Fig. S7). Using this system, we tested whether MSC-EVs can suppress photoreceptor apoptosis. We first activated the BV2 cells with LPS and then moved them to mingle with the 661 W cells. Testing the rate of apoptosis with TUNEL staining, we found MSC-EVs reduced 661 W cell death. We then added MSC-EVs and found that it greatly reduced the percentage of TUNEL-positive 661 W cells (green, TUNEL-positive cell number/DAPI-positive number). After LPS treatment, 61.8 ± 3.1% of the cells stained (LPS-treated BV2 group, *n* = 3, Fig. 7A, B), while after EVs treatment this percentage was 41.9 ± 2.0% per image (LPS-treated BV2 + EVs, *n* = 3, *P* = 0.002, Fig. 7A, B).

As LPS treatment activated microglia which may release inflammatory factors that cause photoreceptor death, we further explored which inflammatory factors were modulated by MSC-EVs. Consistent with what we found in our animal study, using a qPCR test, we found that MSC-EVs enhanced the gene expression of the anti-inflammatory cytokines Arg1 (to 2.3-fold of LPS-treated group, *n* = 3, *P* = 0.008, Fig. 7C), and it reduced that of the transcription factor Nr4a3 (to 0.53 of LPS-treated group, *n* = 4, *P* = 0.016, Fig. 7D). In addition, MSC-EVs reduced the expression of several pro-inflammatory cytokines, including IL-1β (1.8-fold less, *n* = 3, *P* = 0.002), TNF-α (1.6-fold less, *n* = 3, *P* = 0.013) and IL-6 (1.7-fold less, *n* = 3, *P* = 0.005) in the LPS-treated BV2 group (Fig. 7E). It also enhanced the expression of two other anti-inflammatory cytokines, Chil3 (2.3-fold higher, *n* = 3, *P* = 0.006) and Retnla (4.5-fold higher, *n* = 3, *P* = 0.003, Fig. 7F). Thus, MSC-EVs treatment significantly reverses the effects of LPS stimulation and modulated many inflammation-related factors.

### MiR-146a-5p-Nr4a3 axis is involved in inflammation modulation

To further elucidate the molecular mechanisms, we searched for a candidate miRNA that may modulate inflammation in rd10 retina. Based on the top 10 miRNAs found in MSC-EVs, we found more than 10 thousands gene that can be targeted by those miRNAs found by the prediction database (http://mirdb.org/mirdb/index.html). Among them, 3 were also differentially expressed between NS-treated and MSC-EVs-treated rd10 retinas; these were Fgl2, Hipk2 and Galnt2l (Additional file 9: Fig. S8A). These 3 genes were predicted to be regulated by miRNA-21-5p, miRNA-146a-5p and miRNA-let7a-5p (Additional file 9: Fig. S8A). However, none of the three genes were downregulated by MSC-EVs treatment. Since miRNAs interfere with the RNA by binding with mRNAs with complementary sequences [32], we focused on the downregulated genes of miRNA after MSC-EVs treatment. Out of the 40 differentially expressed genes between NS- and MSC-EVs-treated rd10 retina, 24 were also differentially expressed between WT and NS-treated rd10 groups (which has 2100 genes). So we further analyzed the 24 genes. Among these 24 genes, there were 8 that were downregulated by MSC-EVs treatment (Additional file 9: Fig. S8B). Based on this, we predicted a connection between miRNA-21-5p, miRNA-146a-5p and miRNA-let7a-5p with the 8 genes. Bioinformatics analysis predicted that Nr4a3 might be the target gene of miRNA-146a-5p with 2 possible binding sites (Additional file 9: Fig. S8C). As miR-146a-5p level indeed changed during photoreceptor degeneration in rd10 retina [33], we therefore hypothesized that miR-146a-5p regulates the expression of Nr4a3, which then inactivates the NF-κB signaling pathway and inflammation (Additional file 10: Fig. S9).

**Fig. 8:**
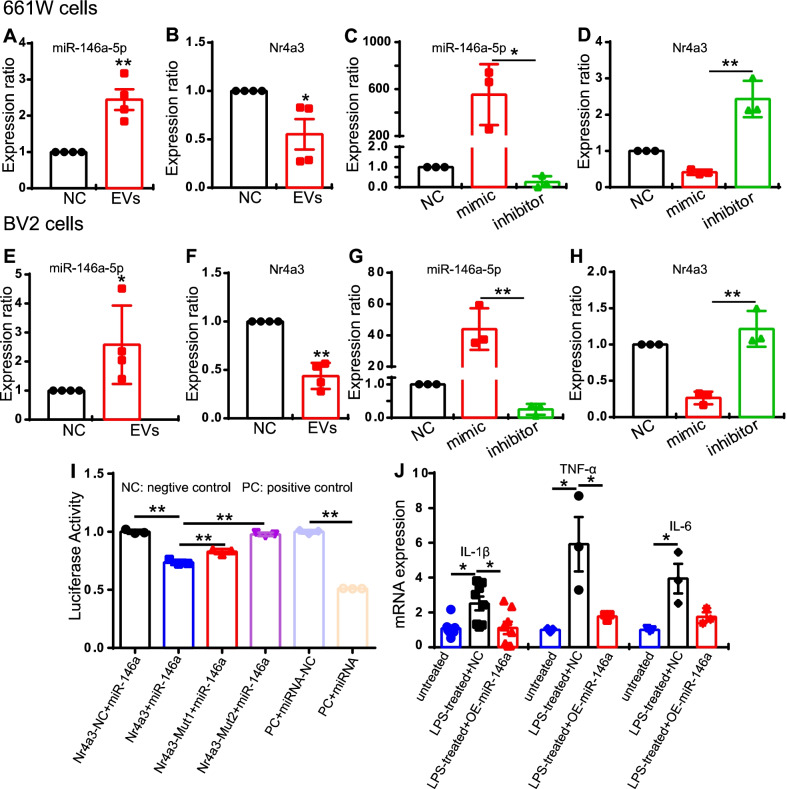
MiR-146a directly regulates the expression of Nr4a3. **A**, **B** The expression of miR-146a-5p (A) and Nr4a3 (B) after MSC-EVs treatment for 24 h in 661 W cells. **C**, **D** The expression of miR-146a-5p (C) and Nr4a3 (D) after overexpressing miR-146a-5p by using the mimic or blocking it using inhibitor in 661 W cells. **E**, **F** The expression of miR-146a-5p(E) and Nr4a3 (F) after MSC-EVs treatment in BV2 cells. **G**, **H** The expression of miR-146a-5p (G) and Nr4a3 (H) after using the mimic or inhibitor in BV2 cells. The expression of Nr4a3 followed the changes of miR-146a-5p. **I** The luciferase activity of miR-146a-5p and Nr4a3 or with two forms mutated at the predicted binding sites of Nr4a3. Values were normalized to that of Nr4a3-negative control. **J** Overexpressing miR-146a in BV2 cells downregulated expression of the LPS-induced pro-inflammatory factors IL-1β, TNF-α and IL-6. Data are shown as mean ± SEM. One-way ANOVA and Student’s t test were applied for statistics. All experiments were repeated for 3 times

To test the relationship between miR-146a-5p and Nr43a-NF-κB, we again used our co-culture system with 661 W and BV2 cells. The associated changes of miR-146a-5p and Nr4a3 were first examined after adding MSC-EVs to the culture medium for 24 h. The expression of miR-146a-5p was significantly upregulated both in the 661 W cells (by 2.44-fold ± 0.28-fold; *n* = 4) and in the BV2 cells (by 2.58-fold ± 0.06-fold, *n* = 4, Fig. 8A, E). At the same time, the expression of Nr4a3 was downregulated after MSC-EVs treatment in both 661 W cells (0.55-fold ± 0.16-fold, *n* = 4, Fig. 8B) and BV2 cells (0.44-fold ± 0.07-fold, *n* = 4, Fig. 8F). Then, we changed the expression level of miR-146a-5p by its mimic or inhibitor and observed its impact on Nr4a3 expression. In both 661 W cells and BV2 cells, overexpression of miR-146a-5 promoted Nr4a3 expression, and downregulating miR-146a-5p by its inhibitor caused an upregulation of Nr4a3 (Fig. 8C, D, G, H). Therefore, miR-146a-5p modulates Nr4a3.

A dual-luciferase reporter experiment was further carried out to examine whether miR-146a-5p directly interacts with Nr4a3. Seeds sequences of miR-146a-5p and Nr4a3, their negative controls (miRNA-NC and Nr4a3-3’UTR-NC) and two mutants of Nr4a3 on the predicted binding sites (Nr4a3-3’UTR Mut1 and Mut2) were tested. One luciferase reporter carried either miR-146a-5p (or its negative control), the other reporter carried either Nr4a3 (or its negative control or mutants) and the luciferase activity was measured. A high luciferase activity indicates a low interaction of two components. The results showed a significant decrease in Luc-Nr4a3-3’UTR + miR-146a (0.74 ± 0.01, *n* = 3, blue bar) group compared to that of Nr4a3-negative control (Luc-Nr4a3-3’UTR-NC + miR-146a) (1.00 ± 0.01, *n* = 3, black bar, *p* = 0.001, Fig. 8I). In contrast, when using either mutant (Mut1 or Mut2), the luciferase activity was significantly increased to 0.83 ± 0.01 for Mut1 (red bar, *p* = 0.007 vs. Nr4a3-miR-146a, *n* = 3, Fig. 8I) and 0.98 ± 0.01 for Mut2 (purple bar, *p* = 0.001 vs. Nr4a3-miR-146a group, *n* = 3, Fig.  8I). As a control, negative control of either Nr4a3(black) or miRNA (light purple, Fig. 8I) showed a high luciferase activity around 1.00, while positive control showed a very low fluorescence (brown bar, Fig. 8I). Therefore, our data indicate that miR-146a-5p directly regulates the expression of Nr4a3. In addition, overexpressing miR-146a-5p by its mimic downregulated the pro-inflammatory cytokines (IL-1β, TNF-TNF-α and IL-6) induced by LPS in BV2 cells (Fig. 8J). Using a qPCR test, we found that overexpressing miR-146a-5p significantly decreased the expression of IL-1β (2.3-fold less, *n* = 8, *P* = 0.02), TNF-α (3.4-fold less, *n* = 3, *P* = 0.05) and a downtrend of IL-6 (2.2-fold less, *n* = 3, *P* = 0.06) in the LPS-treated BV2 group (Fig. 8J).

## Discussion

Stem cell-derived EVs or exosomes, a type of nanoscale biomaterial, carry stem cell active components and have the advantages of having low immunogenicity and being easy to administer, making them a promising vicarious methods of stem cell-based therapy for neurodegenerative retinal diseases, including RP. In our study, we found that administration of MSCs-secreted CD9-, CD63- and CD81-positive EVs is therapeutic in *rd10* mice, a RP mouse model. The therapeutic benefit was observed both on the functional and the morphological levels. Functionally, the visual performance (as observed by optomotor and ERG responses) of the MSC-EVs-treated rd10 mice was significantly better than that of the untreated. Morphologically, the MSC-EVs treatment significantly promoted the survival rate of photoreceptors in vivo. Using TUNEL staining on 661 W photoreceptors cell line that were exposed to activated microglia cells, we showed that photoreceptors survive better after treatment because MSC-EVs significantly inhibited apoptosis. Activated microglia/macrophage cells were observed in NS-treated rd10 retinas by Iba1, F4/80, CD68 and CD206 staining, and the microglia/macrophage cells tended to be inactive after the MSC-EVs treatment. In addition, we found that after treating rd10 mouse retinas with MSC-EVs, NF-κB signaling pathway was upregulated. RNA-seq and qPCR analyses showed that MSC-EVs upregulated anti-inflammatory cytokines while downregulating pro-inflammatory cytokines. MSC-EVs application in vitro decreased the number of TUNEL-positive 661 W cells co-cultured with LPS-stimulated BV2, with similar impact on the cytokine expression as in the in vivo study.

The anti-inflammatory effect by Exos we found was consistent with previous report by Bian et al., 2020 (Ref. 9). Different from their work, our animal model was rd10 mouse. The animal model they used was RCS rat with mutation of MERTK gene, while we used rd10 mouse with a mutation on Pde6b, a mutation that causes RP in humans. Furthermore, the origin of Exos in Bian’s work was derived from neural progenitor cells, and our work was derived from mesenchymal stem cell-derived small EVs. Based on the miRNA-seq, the most expressed miRNAs component was quite different between NPC-Exos and MSC-EVs. Therefore, the molecular mechanism of the protective effect will not be the same in two studies. While Bian’s work did not disclose the detailed anti-inflammation mechanism of EVs, we discovered that miR-146a-Nr4a3 axis plays important role in the protective effect of MSC-EVs in our study. We further demonstrated that miR-146a-5p targeted Nr4a3 in 661 W cells and BV2 cells, and the expression of Nra4a was regulated by miR-146a. Taken together, our results indicate that MSC-EVs preserve the retinal structure and function in rd10 mice mainly via the miR-146a-5p-Nr4a3 axis-mediated inflammation.

### The neuroprotective effect of stem cell-derived EVs/exosomes

Recent studies have reported that different stem cell-derived EVs or exosomes play a neuroprotective role in an animal model of AMD, glaucoma, DR and RD, disorders that lead to irreversible vision loss [9, 14, 15, 20, 34–36]. In these animal models, human umbilical cord blood MSC-Exos [34], mouse adipose tissue MSC-Exos [14], human bone marrow-derived stem cell-derived EVs [20] and mouse neural stem/progenitor cell (NPC)-derived exosomes [9] were reported as the effective components of stem cells which had protective effects. Many of these reported exosomes or EVs countered the injury of retinal neurons or retinal pigment epithelium; they promoted cell survival and recovery of retinal function [9, 20, 36]. In our study, the ONL, where photoreceptors reside, was thicker in the MSC-EVs-treated mice indicating a better photoreceptors’ survival (Fig. 3). This result is similar to those obtained by treating several other models of retinal degeneration with NPC- or MSC-Exos; these include RCS rats [9], MNU-induced photoreceptors’ death and hybridization between rd1 mice and Kunming mice models [36]. Regarding the time of treatment, in our study, we observed improvement of visual sensitivity and visual performance (Fig. 2) after treatment with MSC-EVs. Similar to our study, the improvement of visual function (ERG test) was also reported after NPC-Exos treatment in RCS rat model [9] or after MSC-Exos treatment in an MNU-induced degeneration or after hybridization between rd1 mice and Kunming mice model [36]. In our study, ERG results showed the a-wave and b-wave were increased by MSC-EVs treatment (Fig. 2). Thus, stem cell-derived EVs have a neuroprotective effect in different animal models of retinal diseases.

### The neuroprotective mechanisms of MSC-EVs

Recent studies have also demonstrated the neuroprotective mechanism of stem cell-derived EVs/exosomes. Most studies have reported that anti-inflammation is one of the therapeutic targets of these degenerative diseases. In a retinal laser injury mouse model, Yu et al. suggested that MSC-Exos ameliorated the laser-induced retinal injury and protected retinal neurons partially by downregulating MCP-1 and thus regulating the migration and infiltration of monocytes/macrophages [14]. Zhang et al. found that miR-126 expression in MSC-Exos reduced hyperglycemia-induced retinal inflammation by downregulating the HMGB1 signaling pathway, and MSC-Exos protected retinal neurons by downregulating the expression of NLRP3 inflammasome and NF-κB/P65 in a diabetic rat model [15]. In a photoreceptor degeneration model, Bian et al. demonstrated that NPC-derived exosomes markedly suppressed microglial activation to protect photoreceptors from apoptosis. This occurred because microRNAs (miRNAs) in exosomes suppressed the expression of the pro-inflammatory factors TNF-α, IL-1β and COX-2, which are secreted by microglia. It was suggested that NPC-derived exosomes and their contents may underpin the mechanisms of stem cell therapy in the treatment of retinal degeneration [9]. However, the improvement of visual performance stopped by day 28 after neural progenitor cells-derived Exos treatment. Though the mechanism remains unclear, we think it may be related to dose-dependent and the vitality of the exosomes. In our study, we did notice a decayed therapeutic effect of exosomes when using exosomes stored in − 80 °C for a month time instead of fresh prepared ones. In our study, immunostaining demonstrated patterns of microglial/macrophage activation in the rd10 retinas (Fig. 4). WB results also showed that the NF-κB signaling pathway was involved in MSC-EVs treatment (Fig. 6). In addition, RNA-seq screened 13 genes related to immunity and inflammation among the 40 differentially expressed genes between NS-treated and MSC-EVs-treated rd10 retinas (Fig. 5). Consequently, qPCR tests were carried out, confirming the expression of the anti-inflammatory cytokines Arg1 and Fgl2, which were upregulated after the MSC-EVs-treatment, while the pro-inflammatory cytokines Areg and Nr4a3 were downregulated (Fig. 5). Our findings suggest that exosomal miR-146a-5p-Nr4a3 axis-mediated inflammation may represent a therapeutic approach for treating RP in a clinical setting.

### The effective action of miR-146a and Nr4a3 in inflammation

EVs/Exosomes, which contain lipids, proteins and nucleic acids, are now widely recognized as important mediators in intercellular communication regulating cellular proliferation, migration, organization and phenotypes during development, maintenance and function, injury and disease and aging [37, 38]. Most miRNAs in EVs/exosomes play a major role in regulating important molecules to protect retinal neurons [39]. In a photoreceptor degeneration model, Deng et al. demonstrated that miR-21 originating from MSC-Exos strictly maintains photoreceptor survival against MNU injury by targeting programmed cell death [36]. In an optic nerve crush rat model, Mead et al. found that exosomes protect retinal neurons by exosome delivery, and that the protective effects were dependent on miRNAs for the diminished therapeutic effects of MSC-Exos after knockdown of Argonaute-2 [35]. Thereafter, they identified six candidate miRNAs (miR-26a, miR-30c-2 and miR-92a; miR-292, miR-17 and miR-182) and expressed a combination of three of these candidate miRNAs to treat optic nerve crush injury, finding that these candidate miRNAs promoted RGC survival [40]. Actually, miR-146a has mostly been documented in the context of central nervous system (CNS) diseases, especially in Alzheimer's disease (AD). However, the effective action of miR-146a in inflammation remains unclear. Some researchers have pointed out that miR-146a promoted inflammation via the STAT1/cMyc signaling pathway [41], or by targeting Toll-like receptor, leading to a decrease in Aβ clearance [42]. Others have reported that it targets TP53-induced glycolysis and apoptosis regulators, leading to the upregulation of NF-κB [43]. Finally, some have stated that it triggers oxidative stress by activating MAPK signals, thereby increasing Aβ deposition and aggravating the AD process [44]. In contrast to the above findings, most studies have reported that miR-146a has an anti-inflammatory effect in AD [45–47], autoimmune anterior uveitis [48], intracerebral hemorrhage [49–51], spinal cord injury[52–54], amyotrophic lateral sclerosis [55, 56], depression [57, 58], traumatic brain injury TBI [59], subarachnoid hemorrhage [60], experimental autoimmune encephalomyelitis [61] and diabetic encephalopathy [62]. Thus, the mechanisms of miR-146a action in these CNS diseases include downregulating pro-inflammatory cytokines, depressing the NF-κB signaling pathway and targeting TLR/IRAK/TRAF signaling. In our study, the NF-κB signaling pathway was also involved after enrichment with miR-146a-5p in the context of MSC-EVs treatment (Figs. 6 and 8 , Additional file 8: Fig. S7). However, how miR-146a-5p was involved in the NF-κB signaling pathway remained unclear, so Nr4a3 assessments were carried out.

Nr4a3, also known as NOR-1, is a transcription factor which has been documented on rare occasions in the context of inflammation. It also belongs to the orphan nuclear receptors family, whose endogenous ligands remain unknown [63]. The role of orphan nuclear receptors family members in inflammation and immune responses also remains controversial and unclear. A review reported that N4a3, like all Nr4a receptors, was induced by stressors and upregulated by inflammatory conditions [64]. Downregulating Nr4a3 significantly suppressed the expression of LPS-mediated upregulation of CD80, CD86, IL-10, IL-6 and IL-12 [65]. Furthermore, the expression of IKKβ, IRF4 and IRF8 was significantly decreased in NR4A3 siRNA-introduced bone marrow-derived dendritic cells [65]. Similar reports in osteoarthritis indicated that overexpressing Nr4a3 activated IL-1β-induced NF-κB, and that downregulating Nr4a3 significantly inhibited IL-1β-induced NF-κB [31]. These results suggested Nr4a3 promoted inflammatory reactions, consistent with our results on the expression patterns of IL-1β, NF-κB and pro-inflammatory cytokines by WB and PCR (Figs. 6 and 7). Taken together, results showed that MSC-EVs depress inflammation in rd10 retinas via the miR-146a-5p-Nr4a3 axis signaling pathway.

However, to determine how miR-146a and Nr4a3 regulate inflammation in vivo will require more experiments. For the ongoing experiments, we are now collecting the miR-146a-5p overexpressed exosomes and constructing Nr4a3 knockdown and overexpression plasmids in order to apply them in microglia, Müller glia, macrophages and astrocytes for in vivo and in vitro experiments.

## Conclusions

In summary, our results indicate that MSC-EVs preserve the retinal structure and function in rd10 mice mainly via the miR-146a-5p-Nr4a3 axis-mediated inflammation. It is evident that EVs/exosomes play key roles in the stem cell-based paracrine pathway in the treatment of retinal diseases. Although treatment with EVs/exosomes can prevent the risk factors associated with delivering dividing cells into the eye, details of unidentified mechanisms still need to be explored.

## Supplementary Information

**Additional file 1. Fig. S1**. Experimental methods and protocol. (A) Schematic diagram of intravitreal injection of MSC-EVs. (B) Experimental protocol.

**Additional file 2. **The sequence information of the qRT-PCR primers.

**Additional file 3. Fig. S2**. Uptake of MSC-EVs over time in mouse retinas. At 3 h (A), 6 h (B), 12 h (C) and 24 h (D) after the injection, the uptake of PKH26-labeled MSC-EVs (red) by retinal cells (DAPI, blue) was observed at the retinal ganglion cell layer. As time progressed, the uptake rate gradually increased. The white arrows point to the exosomes absorbed in the retinal cells indicated by the double labeling of PKH26 and DAPI.

**Additional file 4. Fig. S3**. Uptake of MSC-EVs in retinal glial cells. (A, B) Image of a cryo-sectioned eye cups that were stained with DAPI (blue), showing the distribution of PKH26-labeled EVs (red) with squared area enlarged in B. (C) Images of whole-mount retinas of PKH-26 (red fluorescent marker) at the level of ONL, INL and GCL. The fluorescent marker is seen in all retinal layers. (D, E, F) Images of whole-mount retinas stained for GFAP (D the focus is on the ganglion cell layer), GS (E, the focus is on the INL), and Iba1(F, the focus is on the INL and GCL). The somas of Müller Glia are located in INL and the somas of astrocytes are located in GCL. The morphology and the location of astrocytes and Müller Glia are totally different in retinas, so we can easily distinguish between them by focusing on different layers. White arrows point to the somas of Müller glial cells or microglial cells that co-labeled with PKH26 in D and E respectively. Pink arrows point to somas of astrocyte which didn’t uptake MSC-EVs.

**Additional file 5. Fig. S4.** Uptake of MSC-EVs in rd10 mouse retinas. Retinal sections stained with DAPI (blue) showed that there were some Dio-488 (green) and PKH26 (red) labeled EVs in MSC-EVs treated rd10 mice (B) but none were observed in NS-treated rd10 retina (A).

**Additional file 6. Fig. S5**. Functional miRNAs are detected in MSC-EVs. (A) MiRNA-seq results from the MSC-EVs we collected from human umbilical cord, with top ten highly expressed miRNAs listed. (B) KEGG analysis of major signal pathways involved in these top ten miRNAs.

**Additional file 7. Fig. S6**. Identification of BV2 cell line by Iba1 staining. (A, B) Images of BV2 cells stained with DAPI (blue) and Iba1(green, B), with no primary antibody as control (A).

**Additional file 8. Fig. S7**. Identification of 661W cell line by recoverin and R&G opsin staining. (A, B) Images of 661W cells stained with DAPI (blue) and anti-recoverin (green, A), or opsin (red, C), with no primary antibody as a controls (B, D).

**Additional file 9. Fig. S8**. Prediction of the relationship of miR-146a-5p and Nr4a3. (A) From the 10 thousand genes that may be modulated by the top 10 miRNA in MSC-EVs, 3 were found to be also differentially expressed between NS-treated and MSC-EVs treated rd10 retinas, which were predicted to be regulated by miRNA-21-5p, miRNA-146a-5p and miRNA-let7a-5p. (B) From the 40 differentially expressed genes following MSC-EVs treatment, 24 were also differentially expressed between WT and NS-treat rd10 group, among which 8 were downregulated and Nr4a3 was one of them. (C) The predicted binding sites of seed sequence of miR-146a-5p on 3’UTR of the sequence of Nr4a3.

**Additional file 10. Fig. S9**. Schematic diagram of the hypothetical mechanisms of MSC-EVs treatment. During photoreceptor degeneration or LPS induced injury, inflammatory reaction appears, which induces the upregulation of Nr4a3 that activates NF-κB and its downstream signaling pathway, leading to the upregulation of pro-inflammatory cytokines (IL-1β, TNF-α, IL-6, Areg) and secondary photoreceptor death. As feedback, the inflammatory reaction also upregulates miR-146a to inhibit Nr4a3 induced inflammatory response. However, once used up, the miR-146a could not be supplemented immediately. Treatment with MSC-EVs upregulates the expression of miR-146a, therefore, can inhibit the expression of Nra4a and suppress the inflammatory response to promote the photoreceptor survival.

## Data Availability

The datasets used and/or analyzed during the current study are available from the corresponding author on reasonable request.

## Abbreviations

RP: Retinitis pigmentosa
MSCs: Mesenchymal stem cells
MSC-EVs: Mesenchymal stem cell-derived EVs
P14: Postnatal day 14
TUNEL: Terminal deoxynucleotidyl transferase-mediated nick end labeling
Nr4a3: Nuclear receptor subfamily 4, group A, member 3
MiR-146a: MicroRNA-146a-5p
NF-κB: Nuclear factor kappa-B
EVs: Extracellular vesicles
Exos: Exosomes
TNF-α: Tumor necrosis factor-α
IL-1β: Interleukin-1 beta
Pro-IL-1β: Precursor of IL-1β
Areg: Amphiregulin
Fgl2: Fibrinogen-like 2
IL-6: Interleukin-6
DMEM/F-12: Dulbecco’s modified Eagle medium containing nutrient mixture F-12
FBS: Fetal bovine serum
BFGF: Basic fibroblast growth factor
NS: Normal saline
IκBα: Inhibitor of NF-κB
P-IκBα: Phospho-inhibitor of NF-κB
