# Supplementary figures and images for: Mesenchymal stem cell-derived extracellular vesicles protect retina in a mouse model of retinitis pigmentosa by anti-inflammation through miR-146a-Nr4a3 axis
Source: Stem Cell Res Ther. 2022 Aug 3;13:394. doi: 10.1186/s13287-022-03100-x (PMC9351183; doi:10.1186/s13287-022-03100-x)

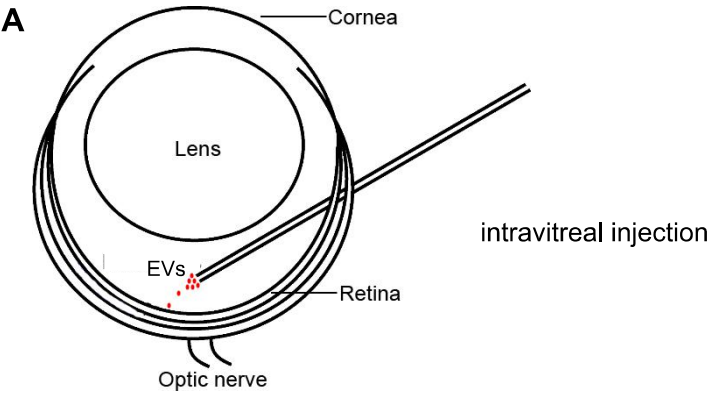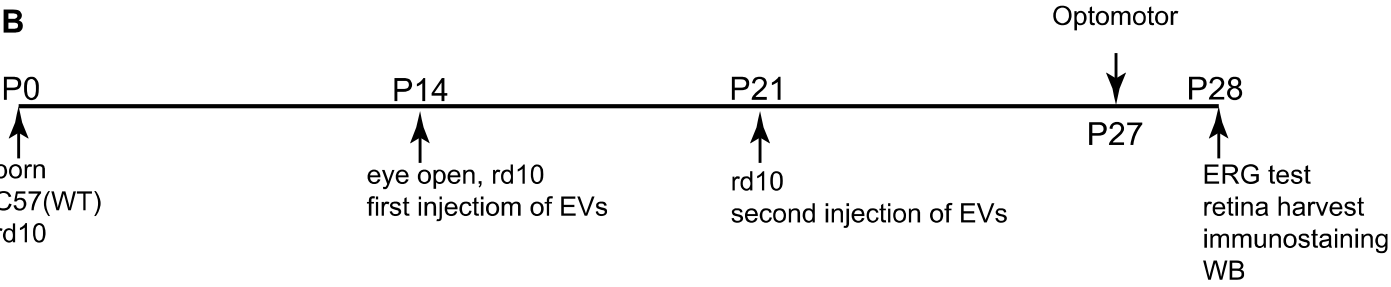

Supplement: Supplementary file 1 — Additional file 1. Fig. S1. Experimental methods and protocol. (A) Schematic diagram of intravitreal injection of MSC-EVs. (B) Experimental protocol. [file 13287_2022_3100_MOESM1_ESM.pdf]

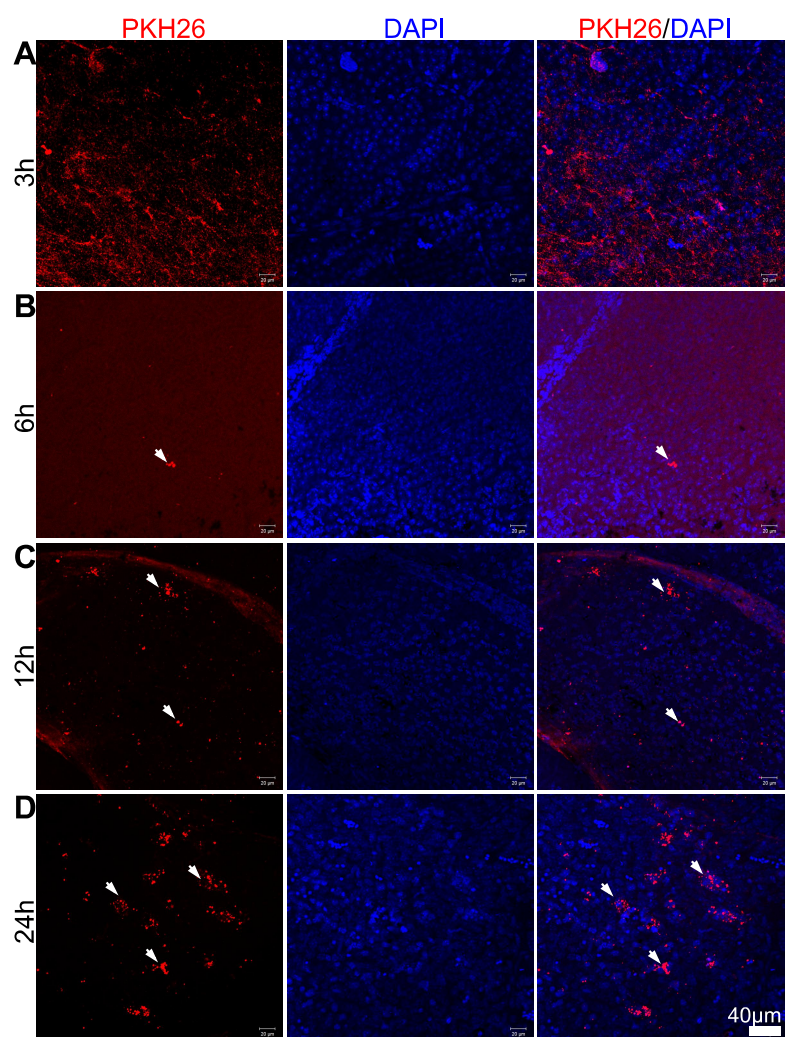

Supplement: Supplementary file 3 — Additional file 3. Fig. S2. Uptake of MSC-EVs over time in mouse retinas. At 3 h (A), 6 h (B), 12 h (C) and 24 h (D) after the injection, the uptake of PKH26-labeled MSC-EVs (red) by retinal cells (DAPI, blue) was observed at the retinal ganglion cell layer. As time progressed, the uptake rate gradually increased. The white arrows point to the exosomes absorbed in the retinal cells indicated by the double labeling of PKH26 and DAPI. [file 13287_2022_3100_MOESM3_ESM.pdf]

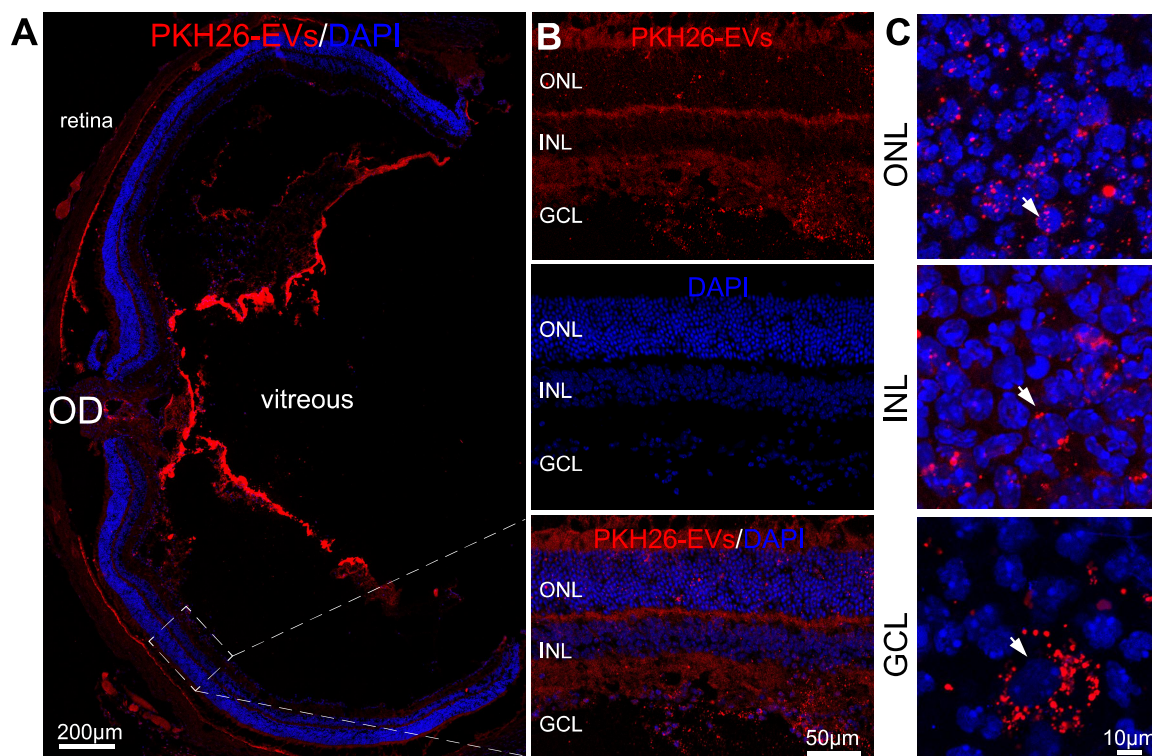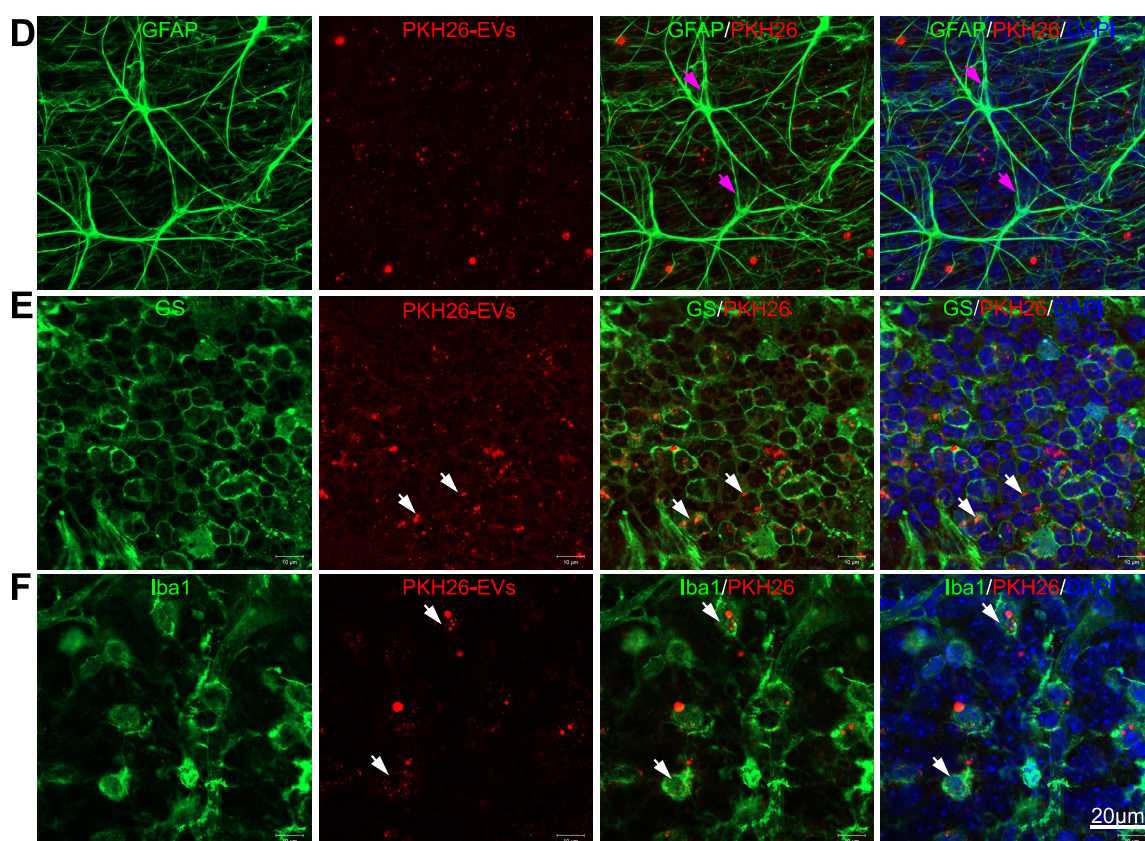

Supplement: Supplementary file 4 — Additional file 4. Fig. S3. Uptake of MSC-EVs in retinal glial cells. (A, B) Image of a cryo-sectioned eye cups that were stained with DAPI (blue), showing the distribution of PKH26-labeled EVs (red) with squared area enlarged in B. (C) Images of whole-mount retinas of PKH-26 (red fluorescent marker) at the level of ONL, INL and GCL. The fluorescent marker is seen in all retinal layers. (D, E, F) Images of whole-mount retinas stained for GFAP (D the focus is on the ganglion cell layer), GS (E, the focus is on the INL), and Iba1(F, the focus is on the INL and GCL). The somas of Müller Glia are located in INL and the somas of astrocytes are located in GCL. The morphology and the location of astrocytes and Müller Glia are totally different in retinas, so we can easily distinguish between them by focusing on different layers. White arrows point to the somas of Müller glial cells or microglial cells that co-labeled with PKH26 in D and E respectively. Pink arrows point to somas of astrocyte which didn’t uptake MSC-EVs. [file 13287_2022_3100_MOESM4_ESM.pdf]

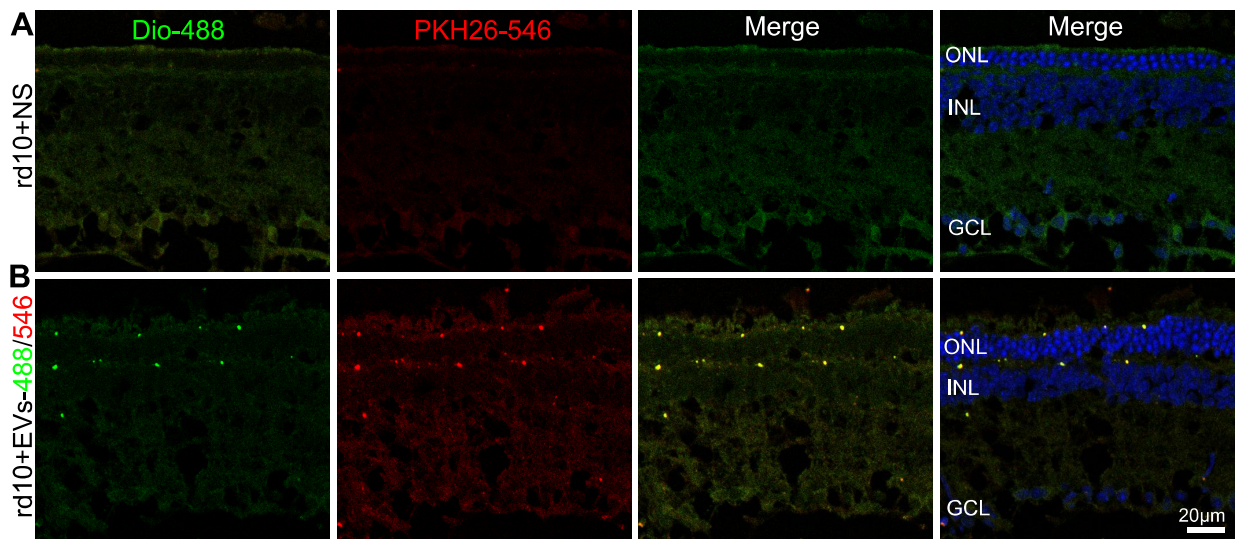

Supplement: Supplementary file 5 — Additional file 5. Fig. S4. Uptake of MSC-EVs in rd10 mouse retinas. Retinal sections stained with DAPI (blue) showed that there were some Dio-488 (green) and PKH26 (red) labeled EVs in MSC-EVs treated rd10 mice (B) but none were observed in NS-treated rd10 retina (A). [file 13287_2022_3100_MOESM5_ESM.pdf]

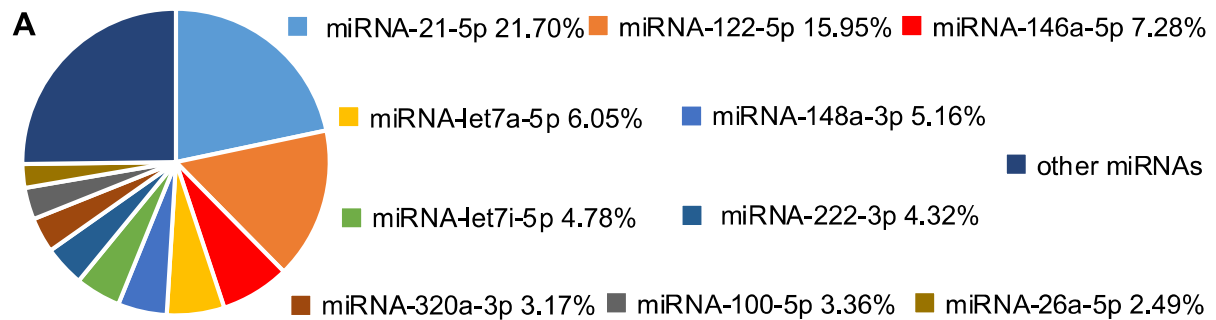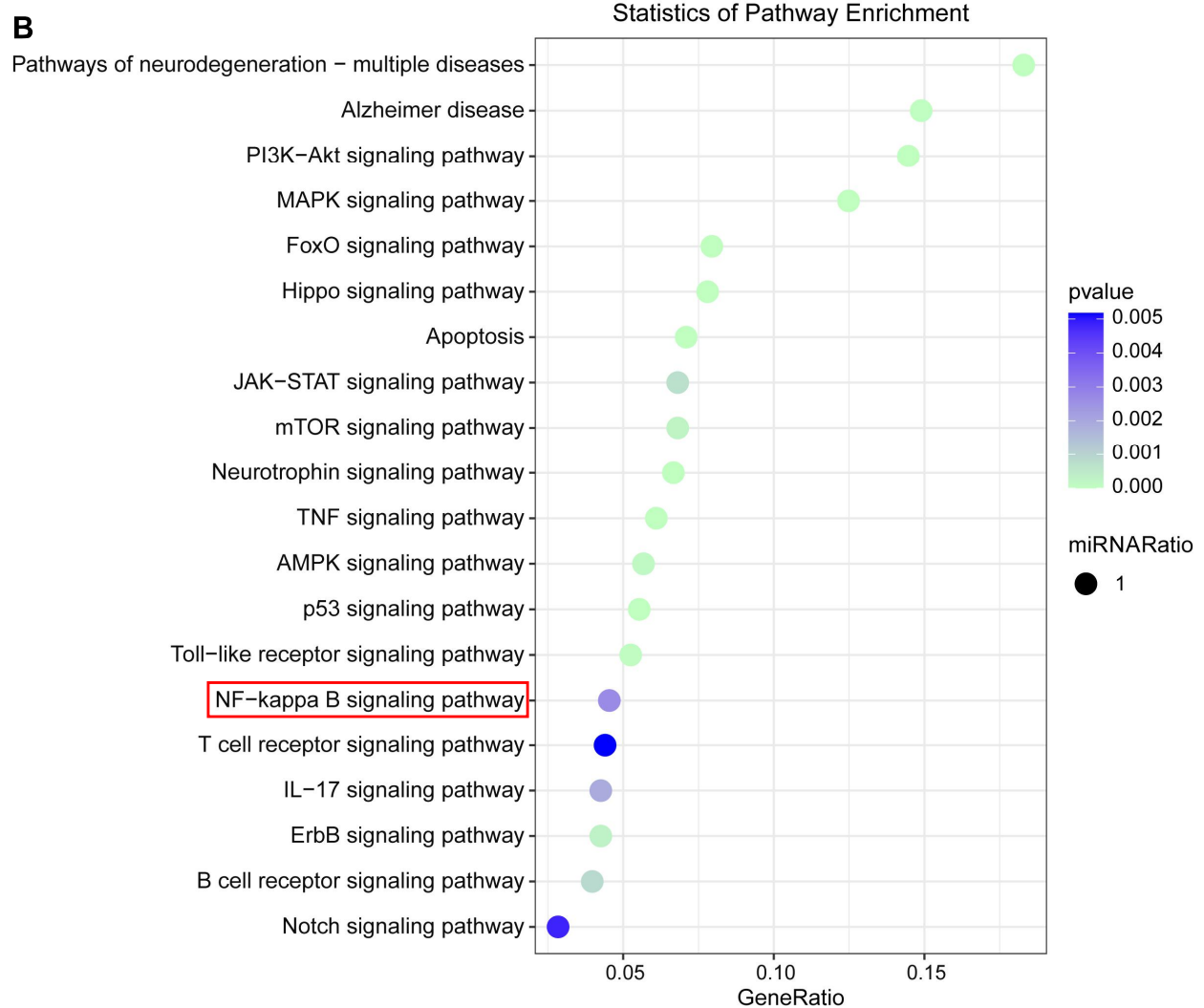

Supplement: Supplementary file 6 — Additional file 6. Fig. S5. Functional miRNAs are detected in MSC-EVs. (A) MiRNA-seq results from the MSC-EVs we collected from human umbilical cord, with top ten highly expressed miRNAs listed. (B) KEGG analysis of major signal pathways involved in these top ten miRNAs. [file 13287_2022_3100_MOESM6_ESM.pdf]

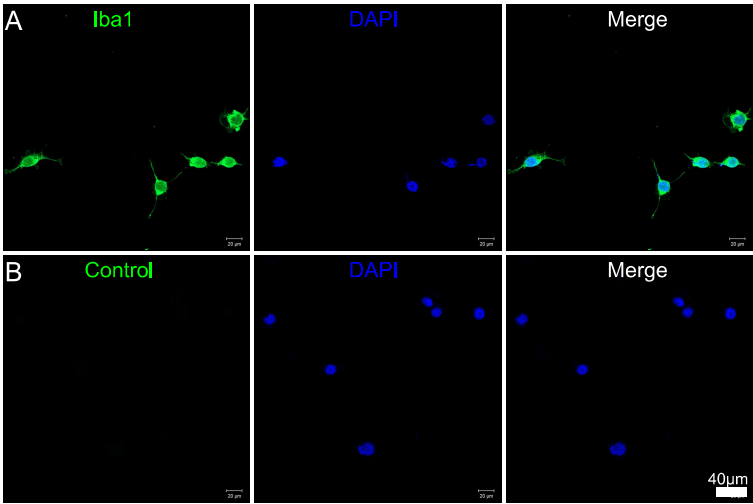

Supplement: Supplementary file 7 — Additional file 7. Fig. S6. Identification of BV2 cell line by Iba1 staining. (A, B) Images of BV2 cells stained with DAPI (blue) and Iba1(green, B), with no primary antibody as control (A). [file 13287_2022_3100_MOESM7_ESM.pdf]

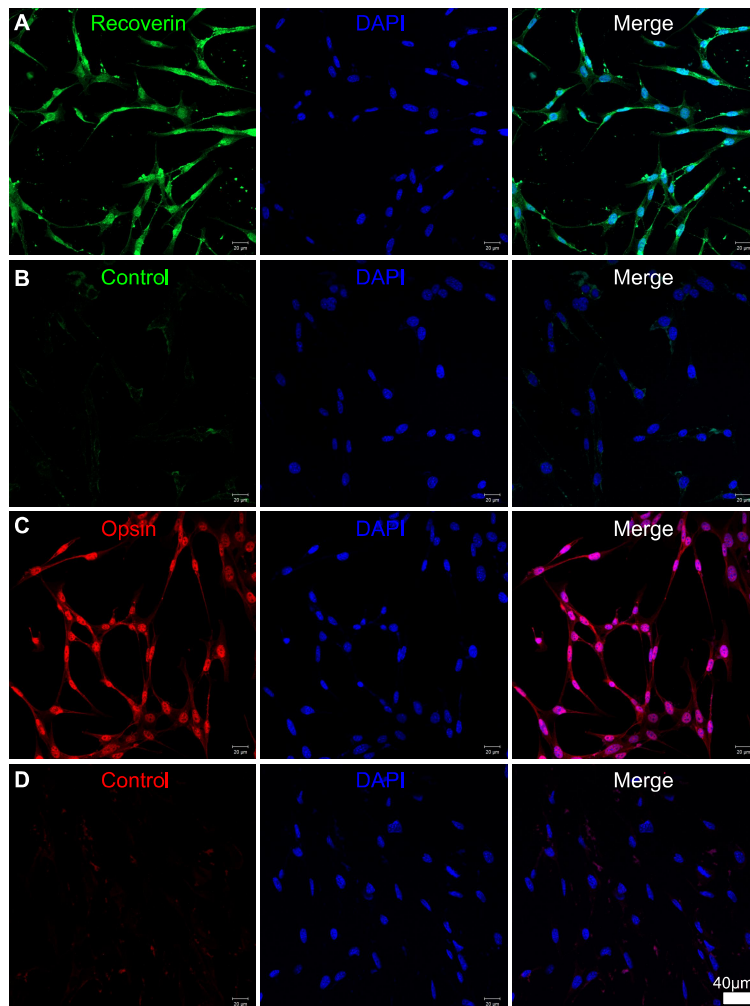

Supplement: Supplementary file 8 — Additional file 8. Fig. S7. Identification of 661W cell line by recoverin and R&G opsin staining. (A, B) Images of 661W cells stained with DAPI (blue) and anti-recoverin (green, A), or opsin (red, C), with no primary antibody as a controls (B, D). [file 13287_2022_3100_MOESM8_ESM.pdf]

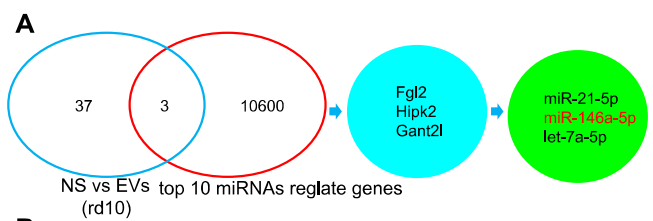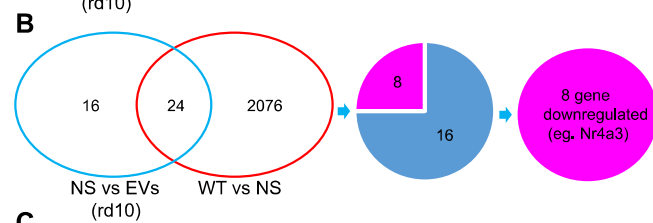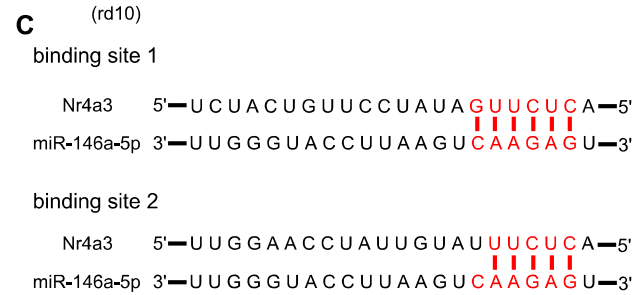

Supplement: Supplementary file 9 — Additional file 9. Fig. S8. Prediction of the relationship of miR-146a-5p and Nr4a3. (A) From the 10 thousand genes that may be modulated by the top 10 miRNA in MSC-EVs, 3 were found to be also differentially expressed between NS-treated and MSC-EVs treated rd10 retinas, which were predicted to be regulated by miRNA-21-5p, miRNA-146a-5p and miRNA-let7a-5p. (B) From the 40 differentially expressed genes following MSC-EVs treatment, 24 were also differentially expressed between WT and NS-treat rd10 group, among which 8 were downregulated and Nr4a3 was one of them. (C) The predicted binding sites of seed sequence of miR-146a-5p on 3’UTR of the sequence of Nr4a3. [file 13287_2022_3100_MOESM9_ESM.pdf]

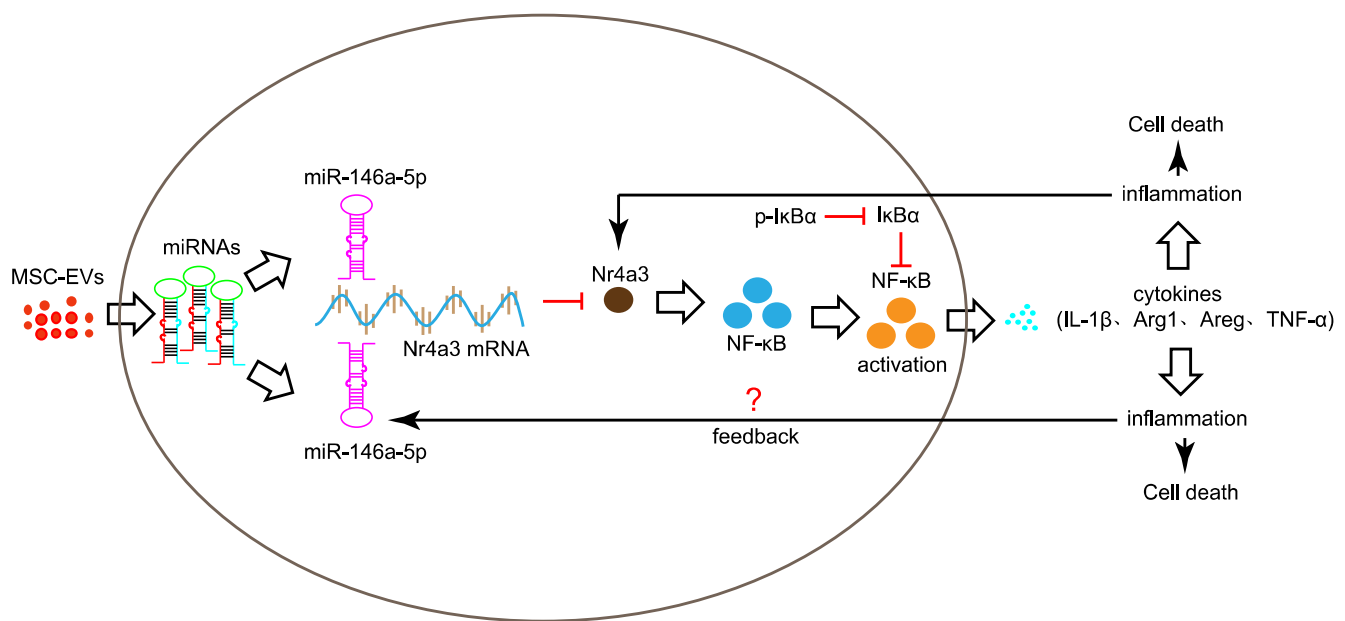

Supplement: Supplementary file 10 — Additional file 10. Fig. S9. Schematic diagram of the hypothetical mechanisms of MSC-EVs treatment. During photoreceptor degeneration or LPS induced injury, inflammatory reaction appears, which induces the upregulation of Nr4a3 that activates NF-κB and its downstream signaling pathway, leading to the upregulation of pro-inflammatory cytokines (IL-1β, TNF-α, IL-6, Areg) and secondary photoreceptor death. As feedback, the inflammatory reaction also upregulates miR-146a to inhibit Nr4a3 induced inflammatory response. However, once used up, the miR-146a could not be supplemented immediately. Treatment with MSC-EVs upregulates the expression of miR-146a, therefore, can inhibit the expression of Nra4a and suppress the inflammatory response to promote the photoreceptor survival. [file 13287_2022_3100_MOESM10_ESM.pdf]
